# Multi-view multi-level contrastive graph convolutional network for cancer subtyping on multi-omics data

**DOI:** 10.1093/bib/bbaf043

**Published:** 2025-02-03

**Authors:** Bo Yang, Chenxi Cui, Meng Wang, Hong Ji, Feiyue Gao

**Affiliations:** School of Computer Science & The Shaanxi Key Laboratory of Clothing Intelligence, Xi'an Polytechnic University, Xi'an 710048, China; School of Computer Science & The Shaanxi Key Laboratory of Clothing Intelligence, Xi'an Polytechnic University, Xi'an 710048, China; School of Computer Science & The Shaanxi Key Laboratory of Clothing Intelligence, Xi'an Polytechnic University, Xi'an 710048, China; School of Computer Science & The Shaanxi Key Laboratory of Clothing Intelligence, Xi'an Polytechnic University, Xi'an 710048, China; School of Computer Science & The Shaanxi Key Laboratory of Clothing Intelligence, Xi'an Polytechnic University, Xi'an 710048, China

**Keywords:** contrastive learning, graph convolutional network, cancer subtype, multi-omics data

## Abstract

Cancer is a highly diverse group of diseases, and each type of cancer can be further divided into various subtypes according to specific characteristics, cellular origins, and molecular markers. Subtyping helps in tailoring treatment and prognosis accuracy. However, the existing studies are more concerned with integrating different omics data to discover potential connections, but ignoring the relationships between consensus information and individual information within each omics level during the integration process. To this end, we propose a novel fusion-free method called multi-view multi-level contrastive graph convolutional network (M$^{2}$CGCN) for cancer subtyping. M$^{2}$CGCN learns multi-level features, i.e. high-level and low-level features, respectively. The low-level features from each view capture the intrinsic information in each omics by reconstruction of node attribute and graph structures. The high-level features achieve cancer subtyping via contrastive learning. Comprehensive experiments were performed on 34 multi-omics cancer datasets. The findings indicate that M$^{2}$CGCN achieves results comparable to or surpassing many state-of-the-art methods.

## Introduction

Cancer is a heterogeneous disease [1–3]. For decades, pathologists have recognized cancer heterogeneity and classified tumors originating from the same organ into distinct histological subtypes [4–6]. Distinguishing cancer subtypes is critical to advancing cancer research since it can improve the quality of patient care and promote the development of personalized cancer treatments. Recent explosive advances in the release of sequencing technology enable us to comprehensively analyze several cancer genome profiles [7–9]. Various significant national and international projects, including The Cancer Genome Atlas (TCGA), have amassed extensive biological samples analyzing multi-level molecular profiles [10, 11], revealing many new cancer genes, pathways, and mechanisms, which would contribute to cancer diagnosis, treatment, and prognosis.

Labeling cancer data is laborious and time-consuming work since it can only via clinical follow-up. Therefore, more and more multi-omics integrative clustering algorithms are employed to achieve cancer subtyping. Early conventional clustering methods, such as K-means [12] and spectral clustering [13], directly integrated each omics data in tandem. Integration-based methods have gradually become mainstream in recent years, starting with CC [14] and PINS [15], where various clustering models are independently trained on each omics data and then the clustering results are fused for ultimate prediction. An alternative approach involves designing a comprehensive representation model to investigate the relationships among different omics data, such as MCCA [16], iCluster [17], MOFA [18], and others. Multi-omics data usually present complex non-linearity, hence some works use kernel trick to some extent deal with nonlinear structure therein data. CIMLR [19] constructs several Gaussian kernels for each omics data, and then combines these kernels to construct one fused similarity matrix. COPS [20] constructs kernel-functions using pathway as prior knowledge and then fuses the different kernels to carry out spectral clustering. IntNMF [21] utilizes the shared factors obtained via non-negative matrix factorization to embed multi-omics data and then to cluster. MCluster-VAEs [22] employs a unified attention-based network architecture to model multi-omics data and leverages a variational Bayes objective function to derive cluster-friendly representations and posterior estimates for clustering assignments. MOCSS [23] constructs two auto-encodes to learning the shared and specific representation, respectively, and applies contrastive learning only to shared representation for clustering. SNF [24] uses message-passing theory to fuse the sample neighborhood graphs constructed on each omics into a unified similarity network. SNFCC [25] combines SNF [24] and CC [14] for clustering. In NEMO [26], per omics dataset, a similarity matrix is constructed and averaged using a kernel based on the radial basis function. In recent years, deep learning algorithms have emerged as a highly promising approach for integrating multi-omics data [27]. These integrative representation models have already achieved satisfactory performance, however, practically, multi-omics data contain consensus information therein all omics levels and meanwhile individual information for omics-specific data. Integration multi-omics could discover the potential clustering patterns, but the integration process usually ignores the individual information which may contain some knowledge that other omics do not have. Therefore, constructing an appropriate fusion-free model is not only convenient for the training procedure but also could promote the subtyping performance.

The primary purpose of clustering is to discover meaningful structures within the data, identify natural groupings, and gain insights into the underlying distribution of the data. Recently, graph neural networks have the ability to uncover patient similarity and complex gene expression patterns from the latent space [28, 29]. Moreover, contrastive learning [30, 31] is a type of self-supervised learning methodology. It is designed to generate data point representations by increasing the similarity among similar data points and decreasing it among dissimilar ones. In the realm of multi-view learning, some recent studies have demonstrated impressive results by employing contrast learning techniques [32, 33]. For instance, Tian and colleagues [32] introduced a contrastive multi-view coding framework aimed at capturing the latent semantics of scenes. Meanwhile, in a separate work [33], the authors devised a contrast-based approach for multi-view representation learning, specifically tailored for addressing graph categorization tasks.

**Figure 1:**
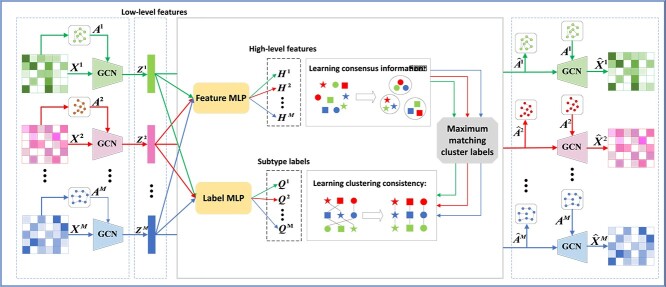
The architecture of M$^{2}$CGCN. First, GCN are applied to extract low-level features from each omics dataset. Next, the feature MLP and label MLP are designed to extract high-level features and predict subtype labels, respectively. The clustering on high-level features assist in improving clustering performance via Maximum matching. Finally, GCN reconstructs node attributes and graph structures while ensuring consistency through contrastive learning.

In this paper, we propose a novel fusion-free multi-omics representation model for cancer subtyping, i.e. multi-view multi-level contrastive graph convolutional network (M$^{2}$CGCN). As shown in Fig. 1, first, the graph convolutional networks are used to learn low-level features contained in each omics data. Second, the overlay feature multilayer perceptron (MLP) and label MLP on the low-level features are designed to obtain high-level features and subtype labels, respectively. Third, in order to improve the effectiveness of clustering, the information obtained by clustering on high-level features with subtype labels is combined. Finally, GCN is used to reconstruct node attributes and graph structures, and meanwhile, the two consistency goals are achieved by comparative learning. To the best of our knowledge, this is the first attempt to construct a fusion-free graph convolutional contrast learning model for multi-omics clustering. The proposed approach achieves results that are comparable to or surpass some state-of-the-art methods across 34 public multi-omics cancer datasets.

## Materials and method

### Datasets

The experiments utilize all 33 datasets from TCGA, encompassing a variety of cancer types. For each type of cancer, we used three omics levels: mRNA expression, DNA methylation, and miRNA expression, for predicting cancer subtypes. In addition, we also performed experiments using mRNA and CNV expression from the Molecular Taxonomy of Breast Cancer International Consortium (METABRIC) breast cancer dataset [34]. The size of the dataset for each cancer subtype is shown in Supplementary Table S5, and the details of datasets are presented in Supplementary Text S1. All data are preprocessed using the same manners as Rappoport et al. [24, 35], details shown in Supplementary Text S2.

### Method

M$^{2}$CGCN includes three modules, i.e. individual encoder, multi-omics contrastive learning, and clustering with high-level features. The details of each module will be explained in the following sections.

#### Individual encoder

A multi-omics dataset $\left \{ \boldsymbol{X}^{m}\in \mathbb{R}^{N\times D_{m}} \right \}_{m=1}^{M}$ includes $N$ samples across $M$ omics levels. $\boldsymbol{x}_{i}^{m}\in \mathbb{R}^{D_{m}}$ denotes the $D_{m}$-dimensional sample from the $m$-th omics data. Let $\left \{ \boldsymbol{A}^{m}\in \mathbb{R}^{N\times N} \right \}_{m=1}^{M}$ denote the corresponding graph structure matrix set. Graph structure $\boldsymbol{A}^{m}$ can be defined as follows:


(1) \begin{align*} & \boldsymbol{A}_{ij}^{m}=\left\{\begin{array}{@{}ll@{}} 1, & if\,\,\boldsymbol{x}_{i}^{m}\in Nei\left( \boldsymbol{x}_{j}^{m} \right) \,\,or\,\,\boldsymbol{x}_{j}^{m}\in Nei\left( \boldsymbol{x}_{i}^{m} \right)\\ 0, & otherwise\end{array}\right.,\end{align*}

where $Nei\left ( \boldsymbol{x}_{j}^{m} \right ) $ denotes the neighbor set of $\boldsymbol{x}_{j}$ in $m$-th omics measurements.

The original omics data $\boldsymbol{X}^{m}$ and the corresponding graph structure matrix $\boldsymbol{A}^{m}$ are inputs of our model. The encoder extracts the representation $\boldsymbol{Z}^{m}$ from $\boldsymbol{X}^{m}$ and $\boldsymbol{A}^{m}$ through convolution operation in two layers of GCN. The $l$-th encoder layer’s outputs are expressed as follows:


(2) \begin{align*} & \boldsymbol{Z}_{l}^{m}=\varphi \left( \left( \boldsymbol{D}^{m} \right)^{{-\frac{1}{2}}}\left( \boldsymbol{A}^{m} \right) ^{\prime}\left( \boldsymbol{D}^{m} \right)^{{-\frac{1}{2}}}\boldsymbol{Z}_{l-1}^{m}\boldsymbol{W}_{l}^{m} \right),\end{align*}

where $\varphi \left ( \cdot \right ) $ denotes a tanh activation function. $\left ( \boldsymbol{A}^{m} \right ) ^{\prime}=\boldsymbol{A}^{m}+\boldsymbol{I}$ and $\boldsymbol{I}$ is the identity matrix, $\boldsymbol{D}_{ii}^{m}=\sum _{j}{\left ( \boldsymbol{A}_{ij}^{m} \right )}^{\prime}$. The parameter matrix $\boldsymbol{W}_{l}^{m}$ is learned by $l$-th layer, where $l=1,2,...,L$ and $L$ is the layer number of the encoder. When $l=1$, set $\boldsymbol{Z}_{0}^{m}=\boldsymbol{X}^{m}$, and when $l=L$, set $\boldsymbol{Z}^{m}=\boldsymbol{Z}_{L}^{m}$, which is the latent embedded representation of $m$-th omics data.

To conduct the reconstruction, the decoder is designed as the reverse of the encoder, thus the decoder also has a two-layer structure. The reconstruction of the omics data and the reconstruction of corresponding structural graph are computed as equation (3) and equation (5), respectively.


(3) \begin{align*} & \hat{\boldsymbol{Z}}_{L}^{m}=\varphi \left( \boldsymbol{Z}_{L}^{m}\hat{\boldsymbol{W}}_{L}^{m} \right), \end{align*}

(4) \begin{align*} & \hat{\boldsymbol{Z}}_{l-1}^{m}=\varphi \left( \left( \boldsymbol{D}^{m} \right)^{-\frac{1}{2}}\left( \boldsymbol{A}^{m} \right) ^{\prime}\left( \boldsymbol{D}^{m} \right)^{-\frac{1}{2}}\hat{\boldsymbol{Z}}_{l}^{m}\hat{\boldsymbol{W}}_{l-1}^{m} \right), \end{align*}

(5) \begin{align*} & \hat{\boldsymbol{A}}^{m}=\varphi \left( \boldsymbol{Z}_{L}^{m}\overset{}{\tilde{\boldsymbol{W}}^{m}}\left( \boldsymbol{Z}_{L}^{m} \right)^{T} \right), \end{align*}

where $\hat{\boldsymbol{W}}_{l}^{m}$ are the decoder model parameters and the parameters $\tilde{\boldsymbol{W}}^{m}$ are defined by the training of the reconfiguration graph $\hat{\boldsymbol{A}}^{m}$.

Then we define a loss of omics data matrix reconstruction and a loss of corresponding graph reconstruction as follows:


(6) \begin{align*} & \mathcal{L}_{F}=\sum_{m=1}^{M}{\Big\| \boldsymbol{X}^{m}-\left. \hat{\boldsymbol{X}}^{m} \Big\|_{F}^{2} \right.}, \end{align*}

(7) \begin{align*} & \mathcal{L}_{G}=\sum_{m=1}^{M}{\Big\| \boldsymbol{A}^{m}-\left. \hat{\boldsymbol{A}}^{m} \Big\|_{F}^{2} \right.}, \end{align*}

Thus, the reconstruction objective of all omics levels is defined as follows:


(8) \begin{align*} & \mathcal{L}_{Z}=\mathcal{L}_{F}+\mathcal{L}_{G}.\end{align*}

#### Multi-omics contrastive learning

We take the latent embedding representation $\boldsymbol{Z}^{m}$ as a low-level feature and learn the high-level features $\left \{ \boldsymbol{H}^{m} \right \}_{m=1}^{M}$ via the feature MLP, and the feature MLP with one layer. We perform reconstruction objectives and consistency objectives in different feature spaces. To avoid model collapse, the representation ability of $\boldsymbol{Z}^{m}$ is maintained in the low-level feature space by equation (8). To further learn the consensus information, contrastive learning is utilized in the high-level feature space to allow $\boldsymbol{H}^{m}$ to further achieve the consistency objective.

Contrastive learning seeks to enhance the similarity between positive pairs (related samples) and reduce the similarity between negative pairs (unrelated samples). In particular, each high-level feature $\boldsymbol{h}_{\,\,i}^{m}$ has a total of $\left ( MN-1 \right ) $ feature pairs, i.e.,$\left \{ \boldsymbol{h}_{\,\,i}^{m},\boldsymbol{h}_{\,\,j}^{n} \right \}_{i,j=1,...,N}^{m,n=1,...,M}$. Regarding the same sample $i$-th, $\left \{ \boldsymbol{h}_{\,\,i}^{m},\boldsymbol{h}_{\,\,i}^{n} \right \}_{n\ne m}$ are constructed as $\left ( M-1 \right ) $ positive feature pairs, i.e. different omics data of the same patient, and the remaining $M\left ( N-1 \right ) $ are all negative pairs. We use the cosine distance measure, suggested by NT-Xent [28], to determine the similarity between two features:


(9) \begin{align*} & d\left(\boldsymbol{h}_{i}^{m},\boldsymbol{h}_{j}^{n}\right) = \frac{\left<\boldsymbol{h}_{i}^{m},\boldsymbol{h}_{j}^{n}\right>}{||\boldsymbol{h}_{i}^{m}||\,\ ||\boldsymbol{h}_{j}^{n}||},\end{align*}

The loss of feature contrast between $\boldsymbol{H}^{\boldsymbol{m}}$ and $\boldsymbol{H}^{\boldsymbol{n}}$ is defined as follows:


(10) \begin{align*} & \ell_{F}^{\left( mn \right)}=-\frac{1}{N}\sum_{i=1}^{N}{\log \frac{e^{d\left( \boldsymbol{h}_{i}^{m},\boldsymbol{h}_{i}^{n} \right) /\tau_{F}}}{\sum\nolimits_{j=1}^{N}{\sum\nolimits_{v=m,n}^{}{e^{d\left( \boldsymbol{h}_{i}^{m},\boldsymbol{h}_{j}^{v} \right) /\tau_{F}}-e^{1/\tau_{F}}}}}},\end{align*}

where $\tau _{F}$ represents the temperature parameter. Then, the cumulative feature contrast loss at all omics levels is defined as:


(11) \begin{align*} & \mathcal{L}_{H}=\frac{1}{2}\sum_{m=1}^{M}{\sum_{n\ne m}{\ell_{F}^{\left( mn \right)}}},\end{align*}

Next, we illustrate how to cluster in a fusion-free model. In detail, a label MLP is superimposed on the low-level features to get a clustering assignment for all the omics data $\left \{ \boldsymbol{Q}^{m}\in \mathbb{R}^{N\times K} \right \}_{m=1}^{M}$. Define $\boldsymbol{q}_{ij}^{m}$ as the probability that the $i$-th sample is the $j$-th category in the $m$-th omics data. $\boldsymbol{Q}_{. j}^{m}$ represents the vector consisting of the probabilities that each sample under $m$-th omics measure belongs to $j$-th category. We set the last layer of the label MLP to softmax to output the probabilities. To obtain robustness of clustering, similar to the objective of obtaining consistency of high-level features, we utilize contrastive learning to achieve clustering consistency. For the $m$-th omics level, the cluster labels have a total of $\left ( MK-1 \right ) $ label pairs, i.e., $\left \{ \boldsymbol{Q}_{. j}^{m},\boldsymbol{Q}_{. k}^{n} \right \}_{j,k=1,...,K}^{m,n=1,...,M}$, which $\left \{ \boldsymbol{Q}_{. j}^{m},\boldsymbol{Q}_{. j}^{n} \right \}_{n\ne m}$ are defined as $\left ( M-1 \right ) $ positive feature pairs and the remaining $M\left ( K-1 \right ) $ are all negative pairs.


(12) \begin{align*} & \ell_{L}^{\left( mn \right)}=-\frac{1}{K}\sum_{j=1}^{k}{\log \frac{e^{d\left( \boldsymbol{Q}_{. j}^{m},\boldsymbol{Q}_{. j}^{n} \right) /\tau_{L}}}{\sum\nolimits_{k=1}^{K}{\sum\nolimits_{v=m,n}^{}{e^{d\left( \boldsymbol{Q}_{. j}^{m},\boldsymbol{Q}_{. k}^{v} \right) /\tau_{L}}-e^{1/\tau_{L}}}}}},\end{align*}

where $\tau _{L}$ represents the temperature parameter, therefore, the clustering-oriented loss can be defined as follows:


(13) \begin{align*} & \mathcal{L}_{Q}={\frac{1}{2}\sum_{m=1}^{M}{\sum_{n\ne m}{\ell_{L}^{\left( mn \right)}}+\sum_{m=1}^{M}{\sum_{j=1}^{K}{\boldsymbol{s}_{j}^{m}\log \boldsymbol{s}_{j}^{m}}}}},\end{align*}

where $\boldsymbol{s}_{j}^{m}= {\frac{1}{N}\sum \nolimits _{i=1}^{N}{\boldsymbol{q}_{ij}^{m}}}$. The first part of equation (13) is used to obtain clustering consistency, while the second part is a regularization term [36] for avoiding overfitting.

#### Clustering with high-level features

To improve the clustering performance, we use $\left \{ \boldsymbol{Q}^{m} \right \}_{m=1}^{M}$ as an anchor point and match it with the clusters obtained by clustering on the high-level feature $\left \{ \boldsymbol{H}^{m} \right \}_{m=1}^{M}$.

First, K-means [12] is unitized on the high-level features to obtain the clustering information for each omics data. In the $m$-th omics data, define $\left \{ \boldsymbol{c}_{k}^{m} \right \}_{k=1}^{K}$ as the center of the $K$ clusters. The clustering label $\boldsymbol{p}^{m}\in \mathbb{R}^{N}$ of all samples can be calculated as follows:


(14) \begin{align*} & \boldsymbol{p}_{i}^{m}=\mathop{arg\min} \limits_{j}\Big\| \left. \boldsymbol{h}_{i}^{m}-\boldsymbol{c}_{j}^{m} \Big\|_{2}^{2} \right.,\end{align*}

Then, let $\boldsymbol{L}^{m}\in \mathbb{R}^{N}$denote the cluster labels for the output of the label MLP, where $\boldsymbol{l}_{i}^{m}=arg\max _{j}\boldsymbol{q}_{ij}^{m}$. It is important to note that the clusters denoted by $\boldsymbol{p}^{m}$ and $\boldsymbol{L}^{m}$ do not correspond. Thus, using $\boldsymbol{L}^{m}$ as an anchor point, $\boldsymbol{p}^{m}$ is modified by the maximum matching formula [37], which can be described as follows:


(16) \begin{gather*} \min_{E^{m}} \boldsymbol{G}^{m}\boldsymbol{E}^{m}, \notag\\ s.t. \sum_{i=1}{\boldsymbol{e}_{ij}^{m}=1},\sum_{j=1}{\boldsymbol{e}_{ij}^{m}=1}, \notag \\ \boldsymbol{e}_{ij}^{m}\in \left\{ 0,1 \right\},i,j=1,2,...,K, \end{gather*}

where $\boldsymbol{E}^{m}\in \left \{ 0,1 \right \}^{K\times K}$ denotes the boolean matrix and $\boldsymbol{G}^{m}\in \mathbb{R}^{K\times K}$ is the cost matrix, which can be formulated as follows:


(16) \begin{align*} & \boldsymbol{G}^{m}=\max_{i,j}\tilde{\boldsymbol{g}}_{ij}^{m}-\tilde{\boldsymbol{G}}^{m}, \end{align*}

(17) \begin{align*} & \tilde{\boldsymbol{g}}_{ij}^{m}=\sum\nolimits_{n=1}^{N}{\mathcal{I} \left[ \boldsymbol{l}_{n}^{m}=i \right]}\mathcal{I} \left[ \boldsymbol{p}_{n}^{m}=j \right], \end{align*}

where $\mathcal{I} \left [ \cdot \right ] $ is the indicator function. Define the modified cluster assignment $\hat{\boldsymbol{P}}_{i}^{m}\in \left \{ 0,1 \right \}^{K}$ of the $i$-th sample as a one-hot vector. The value of the $k$-th element of $\hat{\boldsymbol{p}}_{i}^{m}$ is 1 only if $k$ satisfies $k=k\mathcal{I} \left [ \boldsymbol{e}_{ks}^{m} \right ] \mathcal{I} \left [ \boldsymbol{p}_{i}^{m}=s \right ],k,s\in \left \{ 1,2,...,K \right \} $. We then optimize the model by cross-entropy loss:


(18) \begin{align*}& \mathcal{L}_{C}=-\sum_{m=1}^{M}{\hat{\boldsymbol{P}}^{m}\log \boldsymbol{Q}^{m}},\end{align*}

where $\hat{\boldsymbol{P}}^{m}=\left [ \hat{\boldsymbol{P}}_{1}^{m},\hat{\boldsymbol{P}}_{2}^{m},...,\hat{\boldsymbol{P}}_{N}^{m} \right ] \in \mathbb{R}^{N\times K}$. Next, we define the loss of consensus information as follows:


(19) \begin{align*}& \mathcal{L}_{P}=\mathcal{L}_{H}+\mathcal{L}_{C},\end{align*}

In summary, the overall loss function for M$^{2}$CGCN is expressed as follows:


(20) \begin{align*}& \mathcal{L} =\mathcal{L}_{Z}+\alpha \mathcal{L}_{P}+\beta \mathcal{L}_{Q},\end{align*}

where $\alpha $ and $\beta $ are trade-off parameters.

In the last, the subtype labels of the $i$-th sample are computed as follows:


(21) \begin{align*}& \boldsymbol{y}_{i}=\mathop{arg\max} \limits_{j}\left( {\frac{1}{M}\sum_{m=1}^{M}{\boldsymbol{q}_{ij}^{m}}} \right).\end{align*}

Algorithm 1 summarizes the entire optimization process of M$^{2}$CGCN in detail. A mini-batch gradient descent algorithm is employed throughout the training process to optimize the model, which integrates a GCN encoder and decoder along with feature and label MLPs. The graph for each omics dataset is constructed by equation (1). Next, the low-level features are learned through the GCN encoder, and the reconstruction of node attribute and graph structure is achieved through the GCN decoder. After contrastive learning of multi-omics through equations (11) and (13), the clustering labels derived from the high-level features are adjusted using the maximum matching formula in equation (15). Finally, the model is fine-tuned with the modified clustering labels by equation (20). Supplementary Tables S1 and S2 provide the node counts for each layer of M$^{2}$CGCN. And, we give a real example to show the overall data flow in Supplementary Table S3.




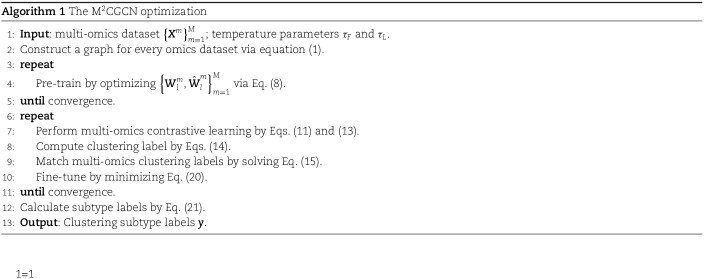



## Result

On 34 cancer datasets, we compared M$^{2}$CGCN with 14 methods, and the comparison methods included directly clustering and integrating methods, i.e. 2 directly clustering methods (K-means [12] and spectral clustering [13]) and 12 integrating methods (LRAcluser[38], CC [14], PINS [15], MCCA [16], iClusterBayes [17], MOFA [18], IntNMF [19], CIMLR [21], MOCSS[23], SNF [24], SNFCC [25], and NEMO [26]).

### Result analysis

The effectiveness of cancer subtyping is evaluated by two metrics, i.e. survival analysis and enrichment analysis on clinical labels. The survival analysis employs the Cox proportional hazards model [39] along with the *P*-value to assess statistically significant differences in survival profiles across different cancer subtypes. For the enrichment analysis of clinical labels, a standardized set of patient clinical details, including gender and age at diagnosis, is utilized alongside four distinct clinical pathological parameters: overall progression (pathologic stage), lymph node involvement (pathologic N), metastases (pathologic M), and tumor progression (pathologic T), is selected for all cancers. The details of the evaluation metrics are described in Supplementary Text S3. To ensure greater adaptability of our model, the number of clusters is treated as an input parameter. This parameter can be determined automatically or set using prior medical knowledge when available. For automatic determination, we concatenate the low-level features $[\boldsymbol{Z}^{1},\boldsymbol{Z}^{2},...,\boldsymbol{Z}^{M}]$, perform clustering, and calculate the silhouette coefficient for varying cluster numbers. The number of clusters corresponding to the highest silhouette score is selected as the optimal value. The silhouette-based selection process is illustrated in Supplementary Figs S1(c)–S34(c), where we show the line plots of silhouette scores for different numbers of clusters.

**Figure 2:**
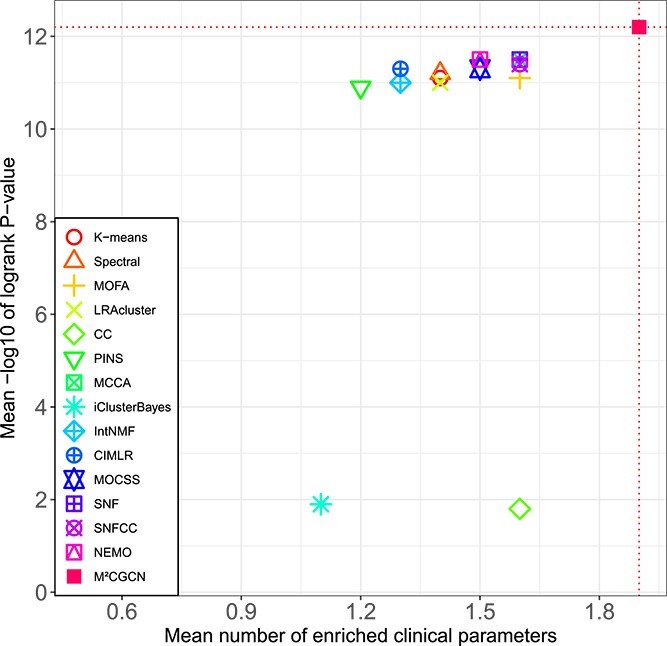
Average performance of various methods across the 34 cancer datasets. The X-axis indicates the enriched clinical parameters’ mean, and the Y-axis displays the -log10 logrank test’s average *P*-values, with the intersection of the red dashed lines highlighting the M$^{2}$CGCN results.

The comparison results on 33 TCGA datasets and METABRIC datasets are shown in Table 1 and Fig. 2. It can be seen that M$^{2}$CGCN achieves the highest average number of enriched clinical parameters and average logrank *P*-value, reaching 1.9 and 12.2, respectively. After M$^{2}$CGCN, five methods achieve a superior average count of enriched clinical parameters at 1.6, while three methods achieve a superior average logrank *P*-value of 11.5. On the COAD dataset, none of the comparative methods identified clusters with significant differences in survival analysis except our method, and our method demonstrated significant survival values in 22 out of 34 datasets. The experimental results indicated that M$^{2}$CGCN outperformed the other 14 comparison methods on 34 cancer datasets, and proved the effectiveness and accuracy of M$^{2}$CGCN in cancer subtyping.

**Table 1:** Clustering result comparison between M$^{2}$CGCN and other approaches on TCGA and METABRIC datasets

Cancer/Alg.	K-Means	Spectral	MOFA	LRAcluste	CC	PINS	MCCA	iClusterBayes	IntNMF	CIMLR	MOCSS	SNF	SNFCC	NEMO	M$^{2}$CGCN
ACC	0/**4.0**(3)	0/**4.2**(4)	1/**5.9**(3)	0/**5.6**(5)	1/**5.4**(3)	0/**6.5**(2)	2/**5.2**(2)	0/**4.4**(4)	0/**3.6**(4)	1/**6.2**(2)	0/**4.3**(4)	2/**4.3**(4)	0/**4.4**(4)	1/**5.1**(3)	1/**5.6**(3)
AML	1/**3.3**(5)	1/**3.2**(6)	1/**3.8**(4)	1/1.3(7)	1/**3.6**(3)	1/**1.5**(4)	1/**1.5**(12)	1/**3.3**(5)	1/**1.9**(5)	0/**1.5**(3)	1/**3.4**(3)	1/**3.1**(6)	1/**4.0**(4)	1/**1.8**(5)	1/**3.0**(5)
BIC	1/**4.6**(4)	1/**5.0**(3)	1/**4.4**(4)	2/**5.2**(5)	1/**2.1**(5)	2/**4.9**(5)	2/**6.3**(5)	1/**4.7**(4)	1/**4.3**(4)	4/**7.4**(13)	1/**5.0**(5)	1/**6.3**(5)	1/**6.5**(5)	2/**6.2**(4)	2/**6.7**(4)
BLCA	5/**2.0**(5)	4/**2.6**(4)	5/**2.8**(5)	5/**2.8**(11)	5/**2.6**(5)	6/1.0(5)	5/**1.6**(10)	0/0.2(4)	6/1.2(5)	5/**3.9**(5)	5/**1.4**(4)	5/**1.9**(3)	5/**3.9**(8)	5/**2.3**(3)	4/**3.0**(3)
CHOL	0/0.5(4)	0/0.2(3)	0/0.2(3)	0/0.3(3)	0/0.1(2)	0/0.1(3)	0/0.4(5)	0/0.3(4)	0/0.3(4)	0/0.1(4)	0/0.2(2)	0/0.1(3)	0/0.1(3)	0/0.3(5)	0/0.3(2)
DLBC	0/0.1(3)	0/0.1(4)	0/0.4(2)	0/0.1(2)	0/0.3(5)	0/0.2(3)	0/0.1(5)	0/0.1(2)	0/0.1(2)	0/0.1(3)	0/0.2(4)	0/0.2(3)	0/0.5(4)	0/0.4(4)	0/0.1(2)
LUSC	0/**1.5**(2)	0/**2.1**(2)	0/**1.7**(2)	0/0.7(12)	1/1.1(4)	0/**2.0**(2)	2/**1.8**(12)	0/**1.9**(5)	0/0.9(3)	1/**1.4**(8)	1/0.9(5)	1/1.3(2)	1/**1.7**(2)	0/**1.8**(2)	2/**2.5**(12)
KIRC	1/0.8(2)	3/**1.4**(3)	1/0.1(2)	3/**1.4**(11)	4/**2.7**(4)	3/**1.7**(6)	3/**6.4**(15)	3/**1.5**(2)	3/0.2(2)	4/1.3(11)	2/0.7(2)	4/**2.1**(4)	4/**2.0**(2)	4/**2.2**(12)	4/**1.5**(2)
HNSC	4/**2.8**(5)	2/**2.1**(4)	4/**2.3**(5)	1/0.6(3)	3/**2.0**(4)	3/**1.7**(5)	3/**1.7**(3)	1/0.5(4)	1/0.3(4)	2/**3.5**(5)	4/**2.0**(5)	2/**2.7**(3)	2/**2.7**(3)	3/**3.0**(4)	3/**3.0**(3)
CESC	1/0.8(4)	2/0.8(4)	1/0.4(2)	1/0.5(3)	1/1.1(3)	1/0.3(4)	1/0.1(3)	1/0.2(4)	1/0.5(3)	1/0.4(3)	3/1.1(3)	2/0.8(4)	0/0.4(5)	2/0.5(5)	3/0.6(4)
KICH	1/0.5(3)	1/0.8(4)	3/0.4(2)	2/0.4(3)	0/0.3(5)	0/0.1(2)	1/0.4(5)	0/0.2(3)	2/0.7(3)	0/0.3(3)	2/0.1(3)	1/0.9(4)	2/0.7(5)	2/1.2(4)	1/1.2(4)
LUAD	1/0.2(2)	1/0.1(3)	1/**3.0**(6)	1/0.1(4)	1/0.4(2)	1/0.3(4)	1/0.2(3)	2/0.1(4)	1/**1.5**(4)	1/0.3(4)	2/0.7(4)	1/**1.6**(3)	1/**2.1**(3)	1/1.0(5)	5/**2.5**(3)
KIRP	5/**7.7**(5)	6/**9.1**(4)	6/**3.6**(4)	5/**7.5**(3)	6/**3.5**(3)	4/**6.3**(4)	5/**5.1**(4)	5/**4.2**(5)	4/**3.8**(5)	4/**2.5**(5)	3/**2.4**(2)	4/**4.5**(3)	6/**3.2**(4)	6/**4.8**(3)	3/**12.1**(5)
ESCA	2/0.1(5)	2/0.3(4)	3/0.1(2)	3/0.1(2)	4/0.3(3)	3/0.6(4)	3/0.1(2)	3/0.1(3)	2/0.1(3)	3/0.1(3)	3/0.1(3)	3/0.1(4)	3/0.1(5)	3/0.1(4)	4/0.2(4)
PAAD	3/**2.5**(4)	3/**2.4**(2)	3/**3.7**(4)	3/**2.6**(8)	3/**3.5**(3)	0/**2.1**(2)	3/**3.0**(6)	0/**1.6**(3)	3/**3.0**(3)	0/1.1(12)	0/**3.2**(2)	3/**3.5**(2)	3/**3.4**(4)	3/**3.5**(3)	4/**4.2**(5)
COAD	1/0.4(2)	1/0.9(12)	1/0.2(2)	1/0.1(10)	0/0.3(2)	2/0.1(4)	0/0.2(2)	1/0.1(2)	1/0.2(3)	2/0.1(11)	2/0.1(2)	0/0.6(3)	2/0.3(10)	0/0.1(3)	1/**1.7**(5)
LIHC	2/0.2(2)	2/0.4(2)	2/0.3(2)	2/**2.9**(12)	1/0.3(2)	2/0.8(5)	2/**1.5**(15)	2/**2.2**(6)	2/**2.0**(5)	3/**2.6**(8)	2/**2.1**(6)	2/**4.2**(5)	2/**3.2**(10)	3/**4.2**(5)	2/**3.2**(5)
OV	0/0.1(2)	2/0.8(4)	0/0.1(2)	1/0.1(4)	1/0.2(3)	1/0.1(2)	1/0.8(9)	1/0.4(6)	0/0.7(3)	1/0.1(2)	1/1.0(3)	1/0.6(3)	1/0.5(3)	1/0.4(3)	1/0.4(4)
UVM	0/**4.7**(4)	0/**3.0**(5)	0/**5.5**(3)	0/**4.0**(3)	0/**5.0**(3)	0/**5.1**(5)	0/**5.0**(3)	0/**4.9**(5)	1/**2.1**(5)	0/**5.7**(5)	0/**5.7**(2)	0/**5.7**(3)	0/**4.7**(3)	0/**3.3**(4)	0/**7.6**(3)
LGG	1/**323**(3)	1/**323**(3)	1/**323**(3)	1/**323**(3)	1/**9.0**(3)	1/**323**(3)	1/**323**(3)	1/**14.0**(3)	1/**323**(3)	1/**323**(3)	1/**323**(3)	1/**323**(3)	1/**323**(3)	1/**323**(3)	1/**323**(3)
MESO	0/**3.3**(4)	0/**2.5**(3)	0/0.6(2)	0/0.9(4)	0/**2.6**(3)	0/**1.7**(5)	0/**1.6**(3)	0/1.1(4)	0/**3.9**(4)	0/**1.7**(4)	0/**2.8**(3)	0/**3.6**(5)	0/**3.9**(3)	0/**2.8**(5)	1/**4.4**(5)
TGCT	3/0.6(3)	1/0.5(4)	3/0.4(2)	2/0.2(3)	2/0.3(2)	1/0.1(4)	2/0.2(3)	3/0.4(4)	1/0.5(4)	0/0.8(4)	3/0.7(3)	3/0.7(3)	2/0.3(2)	3/0.6(3)	2/0.7(3)
UCEC	3/**2.5**(4)	1/**1.5**(5)	3/**2.1**(4)	3/**2.6**(4)	3/**3.9**(3)	2/0.2(5)	3/**3.0**(3)	3/**4.2**(4)	3/**3.7**(4)	3/**3.7**(4)	2/**3.3**(3)	3/**3.4**(5)	3/**3.7**(5)	2/**3.3**(5)	1/0.3(3)
PCPG	0/0.2(4)	0/0.5(5)	0/0.2(4)	0/0.5(5)	0/0.1(3)	0/0.4(3)	0/0.2(3)	0/0.4(5)	0/0.1(5)	0/0.2(5)	0/0.1(5)	1/0.2(3)	0/0.1(4)	0/0.2(4)	1/0.3(5)
PRAD	3/0.2(5)	1/0.1(5)	1/0.3(2)	2/0.1(4)	3/0.2(4)	1/0.1(3)	2/0.4(5)	2/0.1(4)	1/0.4(4)	2/0.6(4)	1/0.1(3)	4/0.1(5)	4/0.2(5)	0/0.1(3)	4/0.2(5)
GBM	2/**2.6**(5)	2/**2.5**(5)	2/**4.2**(5)	2/**1.6**(12)	2/**3.3**(7)	0/0.7(2)	1/**3.6**(11)	2/**2.7**(2)	1/**3.5**(3)	1/**2.9**(8)	1/**4.1**(4)	0/**4.5**(2)	2/**2.6**(9)	1/**3.8**(4)	1/**5.5**(6)
READ	0/0.2(3)	0/0.6(4)	1/0.4(3)	0/0.2(4)	1/0.6(3)	1/0.3(5)	2/0.3(3)	0/0.1(4)	1/0.6(4)	0/0.6(4)	0/0.2(2)	0/0.2(5)	0/0.5(3)	0/0.1(3)	1/0.7(4)
SKCM	2/0.9(2)	3/**1.5**(6)	0/0.5(2)	2/**1.5**(15)	3/**1.5**(4)	2/1.0(15)	2/**4.8**(2)	2/0.6(2)	2/**4.1**(2)	3/**3.3**(4)	1/0.6(4)	1/**1.6**(3)	1/**2.2**(4)	3/**4.0**(5)	3/**4.1**(5)
THYM	1/**1.8**(3)	1/**2.2**(5)	1/**2.9**(3)	1/**1.9**(3)	0/**2.2**(2)	1/**1.5**(4)	2/1.1(4)	1/0.1(5)	1/0.2(4)	0/0.9(4)	1/**1.7**(2)	1/**1.5**(5)	1/1.3(4)	1/0.8(3)	1/**3.5**(4)
STAD	0/**1.4**(5)	1/**1.6**(5)	0/1.1(5)	1/**1.7**(3)	1/1.3(4)	0/**1.4**(3)	0/**3.3**(5)	0/**3.0**(4)	0/0.5(4)	0/**2.3**(4)	1/**3.3**(4)	2/**1.8**(5)	1/1.0(4)	0/**2.1**(5)	2/**1.4**(4)
SARC	2/1.3(2)	2/1.3(2)	2/1.1(2)	2/**2.2**(13)	2/1.0(2)	2/0.8(3)	2/1.0(15)	1/0.9(2)	2/**1.8**(3)	2/**2.5**(5)	2/**2.4**(4)	2/**2.3**(3)	2/**2.0**(3)	2/**1.9**(3)	2/**3.0**(13)
THCA	0/0.5(2)	0/0.6(3)	4/0.2(4)	0/0.4(3)	0/0.2(4)	0/0.7(3)	1/0.3(4)	0/0.4(3)	0/0.3(2)	0/0.6(3)	3/1.2(4)	3/1.1(2)	3/1.2(3)	0/**1.4**(2)	2/**1.6**(2)
UCS	0/0.1(2)	0/0.1(3)	1/0.1(2)	0/0.3(4)	0/0.3(5)	0/0.1(2)	0/0.2(2)	0/0.1(3)	0/1.2(3)	0/0.1(3)	1/0.1(2)	0/0.1(4)	0/0.1(4)	0/0.2(2)	0/0.1(3)
METABRIC	1/1.3(5)	2/**1.8**(7)	2/0.9(5)	1/0.9(7)	2/0.6(6)	1/**1.8**(7)	2/**5.6**(7)	1/**5.7**(7)	1/**3.3**(7)	1/**1.8**(7)	2/**4.6**(9)	2/**2.9**(7)	2/**2.5**(7)	1/**3.5**(9)	2/**5.9**(8)
Mean	1.4/**11.1**	1.4/**11.2**	1.6/**11.1**	1.4/**11**	1.6/**1.8**	1.2/**10.9**	1.6/**11.5**	1.1/**1.9**	1.3/**11**	1.3/**11.3**	1.5/**11.3**	1.6/**11.5**	1.6/**11.4**	1.5/**11.5**	1.9/**12.2**
Sig	22/15	24/18	25/14	24/15	24/15	21/14	26/18	20/14	24/15	20/17	26/17	26/20	25/19	22/20	30/22

*Note*. The results are presented as A/B(C), where A indicates significant clinical parameters identified, B denotes the -log10 *P*-value for survival, and C refers to the number of clusters. A significance threshold of 0.05 is applied, and significant results are shown in bold. Mean represents the average across all datasets, while Sig indicates the count of datasets that display significant outcomes.

**Figure 3:**
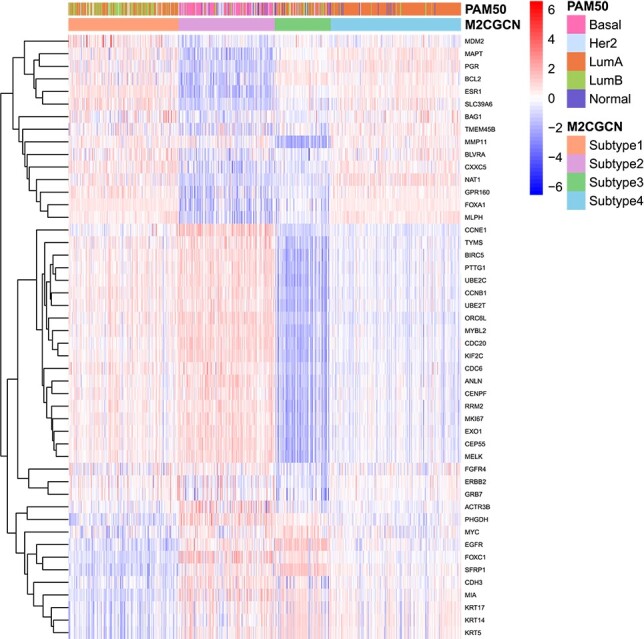
The BIC dataset’s heatmap. Columns denote patients and rows denote mRNAs associated with PAM50. The top column displays the patients’ PAM50 annotation alongside the subtyping results from M$^{2}$CGCN.

To validate the subtyping results produced by M$^{2}$CGCN and compare them with existing subtypes while showcasing the differential expression across distinct subtypes, the experiments are conducted as follows. Initially, the PAM50 classification [40] on the BIC dataset is utilized as a benchmark for comparison. Next, as the PAM50 involves 48 mRNA expression features related to 50 genes, these features are excluded from the original mRNA data of the BIC dataset to eliminate the direct influence of known oncogenes in the multi-omics data. Subsequently, the processed mRNA data, along with other omics data, are used as input for M$^{2}$CGCN. Lastly, a heatmap is generated based on the expression of the 48 mRNA features, highlighting the relationship between oncogenes and the subtypes identified by M$^{2}$CGCN, as well as the overlap between the subtypes identified by M$^{2}$CGCN and PAM50. The heatmap results are shown in Fig. 3, in which patients are rearranged according to subtypes from M$^{2}$CGCN. It is evident that various subtypes exhibit unique expression patterns, and certain subtypes identified by M2CGCN overlap with those from PAM50, such as LumA and our subtype 4, as well as Basal and our subtype 2.

**Figure 4:**
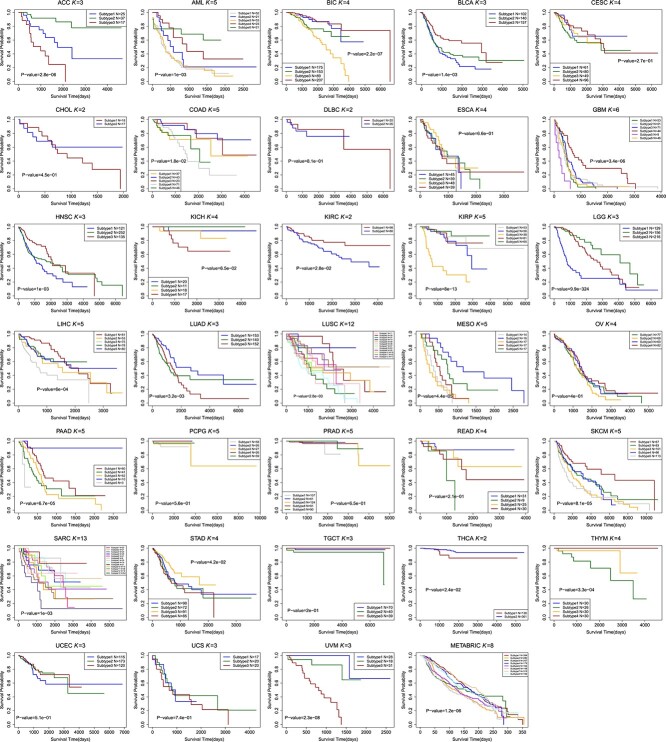
Kaplan–Meier survival curves for 34 cancer types using the M$^{2}$CGCN method. The x-axis represents the number of days since the study began, and the y-axis shows the estimated survival rate.

**Figure 5:**
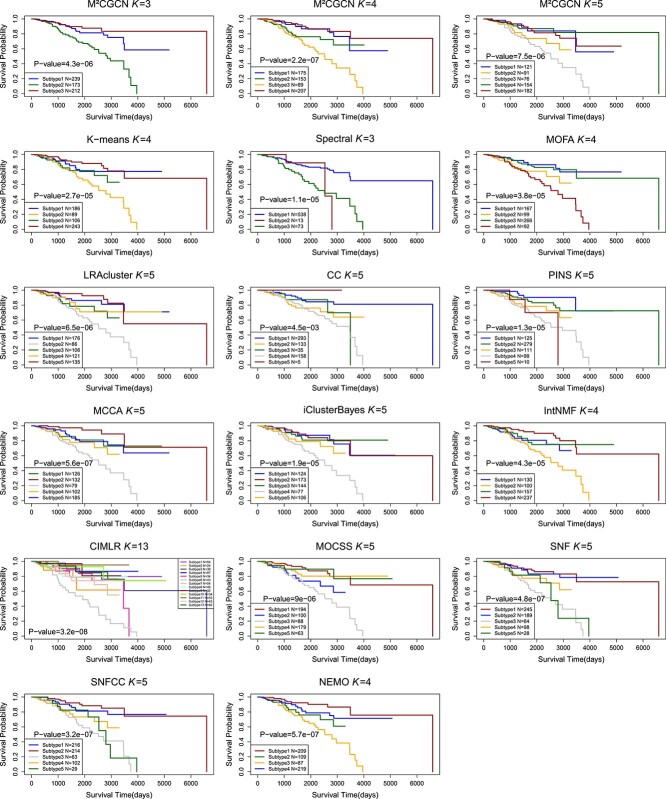
Kaplan–Meier survival curves comparing M$^{2}$CGCN with other methods on the BIC dataset. The x-axis denotes the number of days since the study started, while the y-axis indicates the estimated survival rate.

The Kaplan Meier survival curves of different cancer types using M$^{2}$CGCN are shown in Fig. 4, and the survival curves of BIC using different methods are shown in Fig. 5. In the experiments, the BIC data is grouped into four clusters by the proposed M$^{2}$CGCN. Survival curves for M$^{2}$CGCN are presented using three, four, and five clusters, respectively, to enable direct comparisons with other methods. From these figures we can observe that the M$^{2}$CGCN obtains clearer survival curve separation than other state-of-the-art methods, which illustrates M$^{2}$CGCN is a powerful cancer subtyping method on multi-omics data.

In this paper, experiments on comparing methods are implemented using publicly available code. The optimization is performed by using the Adam optimizer [41], and the M$^{2}$CGCN model is implemented by PyTorch. More details about M$^{2}$CGCN are at https://github.com/chenxi-cui/M2CGCN.

## Model analysis

### Parameter sensitivity analysis

**Figure 6:**
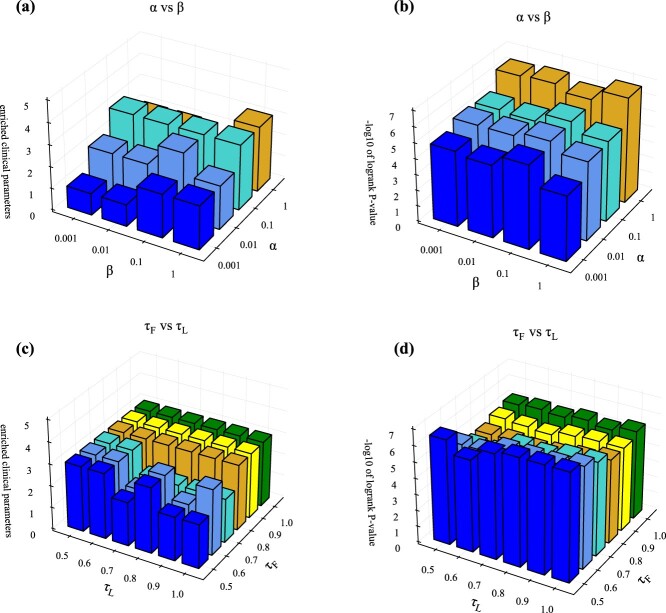
Sensitivity analysis. (a)For M$^{2}$CGCN, enriched clinical parameters are evaluated in relation to $\alpha $ and $\beta $ through sensitivity analysis. (b)For M$^{2}$CGCN, survival analysis is examined relative to $\alpha $ and $\beta $ via sensitivity analysis. (c)For M$^{2}$CGCN, enriched clinical parameters are assessed concerning temperature parameters $\tau _{F}$ and $\tau _{L}$ through sensitivity analysis. (d)For M$^{2}$CGCN, survival analysis is conducted regarding temperature parameters $\tau _{F}$ and $\tau _{L}$ via sensitivity analysis.

There are two hyperparameters $\alpha $ and $\beta $ in equation (20), and we investigated whether hyperparameters are needed to balance the losses in this equation. Figure 6a and b shows the values of the enriched clinical parameters and survival analysis on BIC dataset for different hyperparameters, respectively, demonstrating that our method is not sensitive to $\alpha $ and $\beta $. The reason for this is the well-designed multi-level feature learning framework to reduce the influence between different layers, the trade-off parameters $\alpha $ and $\beta $ are all set equal to 1.0 for simplicity. The other datasets for different hyperparameters are shown in Supplementary Figs S1–S34. In addition, the selection of the two temperature parameters in multi-omics contrast learning needs to be investigated, i.e. $\tau _{F}$ in the high-level feature contrast loss equation (10) and $\tau _{L}$ in the subtype label contrast loss equation (12). Figure 6c and d shows the enriched clinical parameter values and survival analysis values for different $\tau _{F}$ and $\tau _{L}$, respectively, indicating that our method is not sensitive to the choice of $\tau _{F}$ and $\tau _{L}$, which were empirically set to $\tau _{F}=0.5$ and $\tau _{L}=1.0$. We performed sampling at different ratios on the cancer datasets and evaluated the stability of the clustering results [42]. The relevant results are shown in Supplementary Table S4.

### Ablation studies

We performed ablation experiments on the losses in equation (20) to study the contribution of each component individually. Table 2 shows the different loss components and the corresponding experimental results on BIC dataset. In (1) only $\mathcal{L}_{Q}$ is optimized to fulfill the primary objective of multi-omics clustering, which is to capture the cluster consistency. In (2), $\mathcal{L}_{Z}$ is refined to enable low-level features to reconstruct the attribute nodes and associated structural graphs of the multi-omics data. In (3), $\mathcal{L}_{P}$ is refined to extract high-level features and generate clustering labels. (4) is the full loss of M$^{2}$CGCN. The results of (2) and (4) are somewhat better than those of (1) and (3), proving the importance of the reconstruction goal. And the results of (3) and (4) are much better than those of (1) and (2), proving that high-level features are the key contributors to enhancing clustering performance.

**Table 2:** Ablation studies on loss components

	Components			BIC	
	$\mathcal{L}_{Q}$	$\mathcal{L}_{Z}$	$\mathcal{L}_{P}$	Enriched clinical parameters	-log10 logrank test’s *P*-values
(1)	**✓**			1	1.914
(2)	**✓**	**✓**		1	1.959
(3)	**✓**		**✓**	1	4.594
(4)	**✓**	**✓**	**✓**	2	6.659

## Conclusion

The integration of multi-omics data enables researchers and clinicians to capture a more holistic view of cancer biology, as different omics layers provide complementary information about the genetic, epigenetic, and functional changes that occur in cancer cells. By combining these multi-omics data, it becomes possible to identify unique patterns and molecular signatures associated with distinct cancer subtypes. In this paper, we propose M$^{2}$CGCN, a graph convolutional network with contrast learning for predicting cancer subtypes. M$^{2}$CGCN learns low-level features, and high-level features, and on the basis of these multi-level features, obtains cancer subtyping in a fusion-free manner. M$^{2}$CGCN aims to reduce the effect of individual information and better learn the consensus information among different omics data. The experimental findings on public multi-omics datasets from 33 TCGA and METABRIC reveal that M$^{2}$CGCN can achieve advanced performance compared to other relevant methods. Although our study was conducted at two or three omics levels, M$^{2}$CGCN offers a flexible framework that can be readily adapted to handle scenarios involving additional omics data. We believe M$^{2}$CGCN is expected to advance precision oncology and improve patient prognosis.

**Key Points**

- A new unsupervised graph convolutional networks model is proposed to simultaneously learn the cross-omics high level feature representation and cancer subtype labels.
- The contrastive learning is carried out to maintain the consistency of the clustering results from high level representation and the subtype labels from prediction.
- The results of experiments conducted on the TCGA and METABRIC datasets highlight the proposed method’s superior performance in cancer subtype identification.

## Supplementary Material

Supplementary_Materials_bbaf043
