# Supplementary material for: Multi-view multi-level contrastive graph convolutional network for cancer subtyping on multi-omics data
Source: Brief Bioinform. 2025 Feb 3;26(1):bbaf043. doi: 10.1093/bib/bbaf043 (PMC11789786; doi:10.1093/bib/bbaf043)
Supplement: Supplementary_Materials_bbaf043 [file supplementary_materials_bbaf043.pdf]

Supplementary Materials for “Multi-view multi-level contrastive graph convolutional network for cancer subtyping on multi-omics data”

Text S1. Description of the datasets

(1) ACC (Adrenocortical Carcinoma): Adrenocortical Carcinoma is a rare and aggressive malignancy where cancerous cells proliferate within the adrenal cortex and invade surrounding tissues, potentially spreading to other organs.

(2) AML (Acute Myeloid Leukemia): Acute Myeloid Leukemia begins in the bone marrow, rapidly producing abnormal white blood cells. This disrupts the creation of normal cells and can quickly spread into the bloodstream.

(3) BIC (Breast Invasive Carcinoma): Breast Invasive Carcinoma is a type of cancer where malignant cells escape from the milk ducts or lobules and invade nearby breast tissues. Once the cancer has spread beyond its original location, it can potentially travel to other parts of the body via the lymphatic system or bloodstream.

(4) BLCA (Bladder Urothelial Carcinoma): Bladder urothelial carcinoma develops from the cells lining the bladder. It is the predominant form of bladder cancer and can either remain within the bladder lining or progress into invasive stages.

(5) CESC (Cervical Squamous Cell Carcinoma and Endocervical Adenocarcinoma): Cervical cancer is a malignancy that arises from the epithelial cells of the cervix, often associated with human papillomavirus (HPV) infection. Once cancerous cells breach the boundaries of cervical tissue, they may spread via the lymphatic system to the pelvis, lungs, and other regions.

(6) CHOL (Cholangiocarcinoma): Cholangiocarcinoma is a malignancy originating in the bile duct cells, often occurring in intrahepatic or extrahepatic bile ducts. Once the cancer cells invade surrounding tissues beyond the bile duct wall, they may spread through lymphatics or blood vessels to the liver, lymph nodes, and other areas.

(7) COAD (Colon Adenocarcinoma): Colon adenocarcinoma is a malignancy originating from the glandular tissues of the colon. Typically, it develops from non-cancerous polyps which can become cancerous over time.

(8) DLBC (Lymphoid Neoplasm Diffuse Large B-cell Lymphoma): Diffuse Large B-cell Lymphoma is an aggressive form of non-Hodgkin lymphoma that primarily affects lymphatic tissue. Once cancer cells spread within lymph nodes, they may further involve organs like the spleen and bone marrow and eventually enter the bloodstream.

(9) ESCA (Esophageal Carcinoma): Esophageal Carcinoma is a malignancy that develops from the inner lining cells of the esophagus, commonly categorized into squamous cell carcinoma and adenocarcinoma. As the tumor grows, it may penetrate the esophageal wall and invade nearby organs, or metastasize through lymphatic and blood vessels.

(10) GBM (Glioblastoma Multiforme): Glioblastoma Multiforme is a highly aggressive brain cancer that arises from astrocytes. It quickly infiltrates surrounding

brain tissue.

(11) HNSC (Head and Neck Squamous Cell Carcinoma): Head and Neck Squamous Cell Carcinoma often originates in the surface cells of the oral cavity, throat, and nasopharynx. Cancerous cells can spread to nearby muscles, lymph nodes, and metastasize to the lungs and bones through the bloodstream.

(12) KICH (Kidney Chromophobe): Kidney Chromophobe Cancer is a rare form of kidney cancer that primarily arises from the epithelial cells of the kidney's collecting ducts.

(13) KIRC (Kidney Renal Clear Cell Carcinoma): Kidney Renal Clear Cell Carcinoma is the most prevalent type of kidney cancer, primarily affecting the cells that form the lining of the renal tubules responsible for filtering blood.

(14) KIRP (Kidney Renal Papillary Cell Carcinoma): Kidney Renal Papillary Cell Carcinoma is a type of kidney cancer that develops from the epithelial cells of the renal tubules.

(15) LGG (Brain Lower Grade Glioma): Low-Grade Glioma is a slow-growing brain tumor that originates from glial cells. Although it grows slowly, cancer cells can gradually invade critical brain regions and have the potential to progress to high-grade gliomas.

(16) LIHC (Liver Hepatocellular Carcinoma): Hepatocellular carcinoma is the most common form of primary liver cancer, often linked to chronic liver conditions such as cirrhosis or hepatitis B and C infections.

(17) LUAD (Lung Adenocarcinoma): Lung Adenocarcinoma is a cancer that originates from epithelial cells in the lungs, commonly found in the peripheral regions.

(18) LUSC (Lung Squamous Cell Carcinoma): Lung Squamous Cell Carcinoma originates from squamous cells in the lungs. It is commonly associated with smoking and often starts in the central part of the lungs.

(19) MESO (Mesothelioma): Malignant Mesothelioma is a highly aggressive cancer often caused by asbestos exposure, primarily affecting the pleura or peritoneum.

(20) OV (Ovarian Serous Cystadenocarcinoma): Ovarian serous cystadenocarcinoma is a common form of ovarian cancer characterized by the growth of fluid-filled malignant cysts in the ovaries.

(21) PAAD (Pancreatic Adenocarcinoma): Pancreatic adenocarcinoma begins in the cells of the pancreas that produce digestive enzymes. It is known for being particularly aggressive and is often diagnosed at an advanced stage.

(22) PCPG (Pheochromocytoma and Paraganglioma): Pheochromocytoma and Paraganglioma are rare neuroendocrine tumors that usually arise from the adrenal glands or ganglia cells.

(23) PRAD (Prostate Adenocarcinoma): Prostate Adenocarcinoma is a cancer that primarily occurs in the prostate gland, typically growing slowly but, in some cases, invading surrounding tissues and metastasizing to bones and lymph nodes.

(24) READ (Rectum Adenocarcinoma): Rectum Adenocarcinoma is a cancer that originates from epithelial cells in the rectum.

(25) SKCM (Skin Cutaneous Melanoma): Skin cutaneous melanoma is a type of skin cancer that develops in melanocytes, the cells responsible for pigment.

(26) SARC (Sarcoma): Sarcomas are cancers that originate in connective tissues, such as bone, muscle, and fat. They are less common than carcinomas but can occur in various parts of the body.

(27) STAD (Stomach Adenocarcinoma): Stomach Adenocarcinoma is a digestive system cancer that typically originates in the stomach lining.

(28) TGCT (Testicular Germ Cell Tumors): Testicular Germ Cell Tumors are cancers primarily affecting young men and usually originate from germ cells within the testes.

(29) THCA (Thyroid Carcinoma): Thyroid Carcinoma is a cancer that occurs in the thyroid gland, often presenting as a thyroid nodule.

(30) THYM (Thymoma): Thymoma is a rare thoracic tumor originating from the epithelial cells of the thymus.

(31) UCEC (Uterine Corpus Endometrial Carcinoma): Uterine Corpus Endometrial Carcinoma is a common cancer originating in the endometrium, mostly affecting postmenopausal women.

(32) UCS (Uterine Carcinosarcoma): Uterine Carcinosarcoma is a highly aggressive cancer that originates in the endometrium or myometrium.

(33) UVM (Uveal Melanoma): Uveal Melanoma is a malignant tumor originating in the uveal tract of the eye, commonly affecting the iris, ciliary body, or choroid.

(34) METABRIC (Molecular Taxonomy of Breast Cancer International Consortium): The METABRIC dataset is a comprehensive breast cancer resource that aims to categorize the disease by molecular and clinical characteristics. This dataset includes genomic and transcriptomic profiles from over 2,000 patients with breast cancer, which helps reveal different molecular subtypes.

Text S2. Data preprocessing

(1) Removal of outliers, where we remove patient samples with more than 20% missing data in a given histology, and also disregard biological features in patient samples with more than 20% missing values.

(2) Interpolation of missing data, here K nearest neighbours are used to estimate missing data.

(3) Normalization, here we calculate the mean and variance of the biological features, and then subtract the mean and divide by the standard deviation for each feature value to get the normalized feature.

Text S3. Evaluation metrics

We chose survival analysis and enrichment analysis as evaluation metrics. Survival analysis was chosen based on its ability to help us evaluate whether the model successfully identifies groups of patients with different survival durations by comparing the survival curves of different subtypes, thus validating the effectiveness of the model in survival prediction. Meanwhile, survival analysis can also test whether the different clustered subtypes generated by the model are significantly

associated with the survival time of patients, and thus demonstrate whether these subtypes are clinically relevant. The calculation formula is given below:

$$\chi^2 = \sum \frac{(O_i - E_i)^2}{E_i}$$

$$p = 1 - P(\chi^2, df)$$

where O_i is the observed number of deaths and E_i is the expected number of deaths. df is the degrees of freedom and $P(\chi^2, df)$ is the cumulative probability of the chi-square distribution.

Enrichment analysis, on the other hand, is used to assess whether the features or subtypes identified by the model are significantly enriched in certain clinical variables, such as age and pathological stage. This not only helps to explain the correlation between the clustering labels generated by the model and the clinical features, but also provides in-depth clinical interpretation of the model results, thus increasing the interpretability and biological significance of the model. The calculation formula is:

$$H = \frac{12}{N(N+1)} \sum \frac{R_i^2}{n_i} - 3(N+1)$$

This formula is used for continuous variables such as age, where R_i is the rank sum of class i, n_i is the number of samples in class i, and N is the total number of samples.

$$\chi^2 = \sum \frac{(O_i - E_i)^2}{E_i}$$

The formula is used for categorical variables such as gender and pathological staging. Where O_i is the observed value and E_i is the expected value.

Table S1. The number of nodes for Graph Convolutional Encoder, Feature MLP and Label MLP on 33 TCGA cancer datasets.

		ACC	AML	BIC	BLCA	CESC	CHOL	COAD	DLBC	ESCA	GBM	HNSC	KICH	KIRC	KIRP	LIHC	LUAD	LUSC
	N_I	2000	2000	2000	2000	2000	2000	2000	2000	2000	2000	2000	2000	2000	2000	2000	2000	2000
	N_E	1800	1800	1800	1800	1800	1800	1800	1800	1800	1800	1800	1800	1800	1800	1800	1800	1800
	N_R	512	512	512	512	512	512	512	512	512	512	512	512	512	512	512	512	512
mRNA	N_H	256	256	256	256	256	256	256	256	256	256	256	256	256	256	256	256	256
	N_Q	3	5	4	3	4	2	5	2	4	6	3	4	2	5	5	3	12
	N_D	1800	1800	1800	1800	1800	1800	1800	1800	1800	1800	1800	1800	1800	1800	1800	1800	1800
	N_O	2000	2000	2000	2000	2000	2000	2000	2000	2000	2000	2000	2000	2000	2000	2000	2000	2000

	N_I	2000	2000	2000	2000	2000	2000	2000	2000	2000	2000	2000	2000	2000	2000	2000	2000	2000
	N_E	1800	1800	1800	1800	1800	1800	1800	1800	1800	1800	1800	1800	1800	1800	1800	1800	1800
	N_R	512	512	512	512	512	512	512	512	512	512	512	512	512	512	512	512	512
methylation	N_H	256	256	256	256	256	256	256	256	256	256	256	256	256	256	256	256	256
	N_Q	3	5	4	3	4	2	5	2	4	6	3	4	2	5	5	3	12
	N_D	1800	1800	1800	1800	1800	1800	1800	1800	1800	1800	1800	1800	1800	1800	1800	1800	1800
	N_O	2000	2000	2000	2000	2000	2000	2000	2000	2000	2000	2000	2000	2000	2000	2000	2000	2000
	N_I	1000	558	885	383	1000	1000	613	1000	1000	534	1000	1000	791	1000	852	1000	881
	N_E	1800	1800	1800	1800	1800	1800	1800	1800	1800	1800	1800	1800	1800	1800	1800	1800	1800
	N_R	512	512	512	512	512	512	512	512	512	512	512	512	512	512	512	512	512
miRNA	N_H	256	256	256	256	256	256	256	256	256	256	256	256	256	256	256	256	256
	N_Q	3	5	4	3	4	2	5	2	4	6	3	4	2	5	5	3	12
	N_D	1800	1800	1800	1800	1800	1800	1800	1800	1800	1800	1800	1800	1800	1800	1800	1800	1800
	N_O	1000	558	885	383	1000	1000	613	1000	1000	534	1000	1000	791	1000	852	1000	881
		MESO	OV	PAAD	PCPG	PRAD	READ	SKCM	SARC	STAD	TGCT	THCA	THYM	UCEC	UCS	UVM	MESO	
	N_I	2000	2000	2000	2000	2000	2000	2000	2000	2000	2000	2000	2000	2000	2000	2000	2000	
	N_E	1800	1800	1800	1800	1800	1800	1800	1800	1800	1800	1800	1800	1800	1800	1800	1800	
	N_R	512	512	512	512	512	512	512	512	512	512	512	512	512	512	512	512	
mRNA	N_H	256	256	256	256	256	256	256	256	256	256	256	256	256	256	256	256	
	N_Q	5	4	2	5	5	4	5	13	4	3	2	4	3	3	3	5	
	N_D	1800	1800	1800	1800	1800	1800	1800	1800	1800	1800	1800	1800	1800	1800	1800	1800	
	N_O	2000	2000	2000	2000	2000	2000	2000	2000	2000	2000	2000	2000	2000	2000	2000	2000	
	N_I	2000	2000	2000	2000	2000	2000	2000	2000	2000	2000	2000	2000	2000	2000	2000	2000	
	N_E	1800	1800	1800	1800	1800	1800	1800	1800	1800	1800	1800	1800	1800	1800	1800	1800	
	N_R	512	512	512	512	512	512	512	512	512	512	512	512	512	512	512	512	
methylation	N_H	256	256	256	256	256	256	256	256	256	256	256	256	256	256	256	256	
	N_Q	5	4	2	5	5	4	5	13	4	3	2	4	3	3	3	5	
	N_D	1800	1800	1800	1800	1800	1800	1800	1800	1800	1800	1800	1800	1800	1800	1800	1800	
	N_O	2000	2000	2000	2000	2000	2000	2000	2000	2000	2000	2000	2000	2000	2000	2000	2000	
	N_I	1000	616	383	1000	1000	1000	901	838	1000	1000	1000	1000	1000	1000	1000	1000	
	N_E	1800	1800	1800	1800	1800	1800	1800	1800	1800	1800	1800	1800	1800	1800	1800	1800	
	N_R	512	512	512	512	512	512	512	512	512	512	512	512	512	512	512	512	
miRNA	N_H	256	256	256	256	256	256	256	256	256	256	256	256	256	256	256	256	
	N_Q	5	4	2	5	5	4	5	13	4	3	2	4	3	3	3	5	
	N_D	1800	1800	1800	1800	1800	1800	1800	1800	1800	1800	1800	1800	1800	1800	1800	1800	
	N_O	1000	616	383	1000	1000	1000	901	838	1000	1000	1000	1000	1000	1000	1000	1000	

Table S2. The number of nodes for Graph Convolutional Encoder, Feature MLP and Label MLP on METABRIC.

		METABRIC
mRNA	N_I	2000
	N_E	1800
	N_R	512
	N_H	256
	N_Q	8
	N_D	1800
	N_O	2000
CNV	N_I	2000
	N_E	1800
	N_R	512
	N_H	256
	N_Q	8
	N_D	1800
	N_O	2000

Note: M^2CGCN has a 7-layer structure, i.e., input layer, encoder layer, representation layer, feature MLP layer, label MLP layer, decoder layer, and output layer. The number of nodes in each layer is denoted as N_I, N_E, N_R, N_H, N_Q, N_D, N_O respectively. Where the feature MLP layer is an MLP without hidden layer and activation function; the label MLP layer is an MLP without hidden layers, equipped with a softmax activation function, which converts the output of the linear combination of feature into a probability distribution of categories. After the feature MLP gets the high-level features, it calculates the error using the loss function of contrast learning and updates the weights of this network by backpropagation. After the label MLP gets the predicted labels, it first calculates the errors using the loss function for contrast learning and updates the weights of this network by backpropagation. For fine-tuning, the difference between the predicted labels and the pseudo-labels obtained from high-level feature clustering is calculated using cross entropy, and the weights of the feature MLP and the label MLP are updated through backpropagation. The entire network is implemented using the PyTorch framework and the model weights are updated using the Adam optimiser.

Table S3. Data flow using the BIC dataset as an example.

Stage	Processing Step
Data Input	Read pre-processed BIC datasets $\boldsymbol{X}^m$ of different omics (genes, methylation, miRNA).
k-NN Graph Calculation	Compute the k-nearest neighbor (k-NN) graph $\boldsymbol{A}^m$ for each omic, representing local relationships between samples.
Feature Extraction	Extract features from each omic through the encoder, yielding low-level feature $\boldsymbol{Z}^m$.
Contrastive Learning	The feature MLP transforms $\boldsymbol{Z}^m$ into $\boldsymbol{H}^m$, and contrastive loss encourages similar samples to be close in feature space and dissimilar samples to be farther apart.
Classification Task	Low-level feature $\boldsymbol{Z}^m$ is projected into the class space through the label MLP, with Softmax outputting classification probabilities $\boldsymbol{Q}^m$. For fine-tuning, the difference between the predicted labels $\boldsymbol{Q}^m$ and the pseudo-labels $\hat{\boldsymbol{P}}^m$ obtained from high-level feature clustering is calculated using cross-entropy.
Feature Reconstruction	The decoder reconstructs the low-level feature $\boldsymbol{Z}^m$ back to the shape of the input data.
Loss Calculation	Compute contrastive learning loss, classification loss, and reconstruction loss.
Weight Update	Update weights of the encoder, feature MLP, label MLP, and decoder through backpropagation.

Table S4. The results of M^2CGCN at different sampling rates on 34 cancer datasets.

Cancer/θ	0.9	0.7	0.5	0.3
ACC	1/4.9	1/4.7	1/4.1	1/3.9
AML	1/3.0	1/3.2	1/2.6	1/2.3
BIC	2/5.9	2/5.9	2/5.3	1/4.6
BLCA	4/2.8	3/2.3	3/2.5	4/2.5
CESC	3/0.4	2/0.2	2/0.3	1/0.2
CHOL	0/0.3	0/0.2	0/0.2	0/0.1
COAD	2/1.4	1/1.7	1/1.4	1/1.6
DLBC	0/0.1	0/0.1	0/0.3	0/0.1
ESCA	4/0.3	3/0.1	3/0.1	2/0.1
GBM	1/4.1	1/4.3	1/4.7	1/4.0
HNSC	3/2.6	3/2.1	3/2.1	2/2.3
KICH	1/0.9	1/0.8	1/1.1	1/0.5
KIRC	4/1.4	2/1.5	2/1.8	2/1.3
KIRP	3/10.2	2/9.5	2/9.5	1/9.0
LGG	1/323	1/323	1/323	1/323
LIHC	2/2.2	2/1.8	1/1.4	2/1.9
LUAD	5/2.0	4/1.5	2/1.6	2/1.3
LUSC	2/2.3	2/1.8	2/1.4	1/1.7
MESO	1/3.6	1/3	1/2.7	1/2.5
OV	1/0.3	1/0.4	1/0.5	2/0.6
PAAD	4/3.5	3/3.2	3/2.4	2/2.1
PCPG	1/0.2	1/0.4	1/0.3	1/0.3
PRAD	4/0.2	2/0.2	2/0.1	2/0.1
READ	1/0.5	1/0.3	1/0.2	1/0.3
SKCM	3/3.6	3/3.7	3/2.1	1/2.3
SARC	2/2.9	2/2.5	2/3.1	1/2.7
STAD	2/1.2	2/0.7	1/0.5	1/0.4
TGCT	2/0.5	1/0.6	1/0.6	1/0.2
THCA	2/1.3	1/0.7	1/0.9	1/0.5
THYM	1/2.6	1/2.4	1/2.8	1/2.3
UCEC	1/0.4	1/0.1	1/0.3	1/0.1
UCS	0/0.1	0/0.2	0/0.1	0/0.1
UVM	0/6.2	0/5.4	0/5.1	0/4.9
METABRIC	1/5.4	1/3.3	1/4.1	1/4.3

Note: θ is the rate of sampling. In each cell A/B, A is significant clinical parameters detected. B is -log10 P-value for survival.

Table S5. The size of multi-omics datasets.

Dataset	ACC	AML	BIC	BLCA	CESC	CHOL	COAD	DLBC	ESCA	GBM	HNSC	KICH	KIRC	KIRP	LGG	LIHC	LUAD
Size	79	170	624	399	286	35	220	46	171	274	508	66	184	273	501	367	445
Dataset	LUSC	MESO	OV	PAAD	PCPG	PRAD	READ	SKCM	SARC	STAD	TGCT	THCA	THYM	UCEC	UCS	UVM	METABRIC
Size	341	81	287	176	176	473	95	450	257	346	149	499	116	408	57	77	1904

Figure S1. ACC-Adrenocortical Carcinoma (a)Sensitivity analysis of enriched clinical parameters for M2CGCN with respect to α and β. (b)Sensitivity analysis of survival analysis for M2CGCN with respect to α and β. (c)Silhouette score (y-axis) for different number of clusters (x-axis).

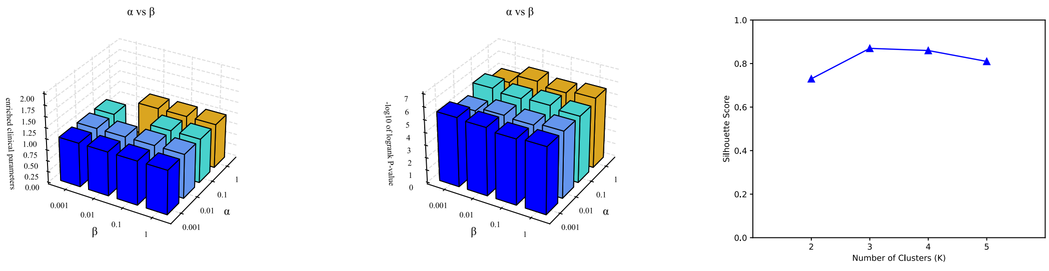

(a) (b) (c)

Figure S2. AML-Acute Myeloid Leukemia (a)Sensitivity analysis of enriched clinical parameters for M2CGCN with respect to α and β. (b)Sensitivity analysis of survival analysis for M2CGCN with respect to α and β. (c)Silhouette score (y-axis) for different number of clusters (x-axis).

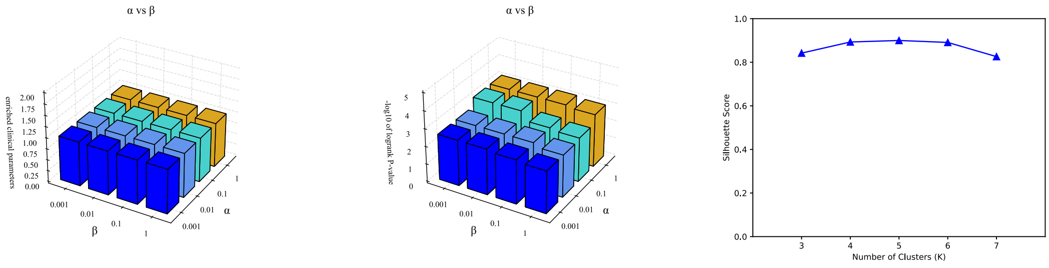

(a) (b) (c)

Figure S3. BIC-Breast Invasive Carcinoma (a)Sensitivity analysis of enriched clinical parameters for M2CGCN with respect to α and β. (b)Sensitivity analysis of survival analysis for M2CGCN with respect to α and β. (c)Silhouette score (y-axis) for different number of clusters (x-axis).

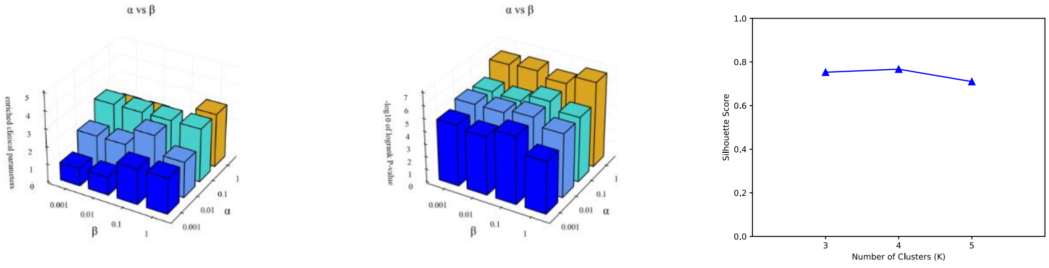

(a) (b) (c)

Figure S4. BLCA-Bladder Urothelial Carcinoma (a)Sensitivity analysis of enriched clinical parameters for M2CGCN with respect to α and β. (b)Sensitivity analysis of survival analysis for M2CGCN with respect to α and β. (c)Silhouette score (y-axis) for different number of clusters (x-axis).

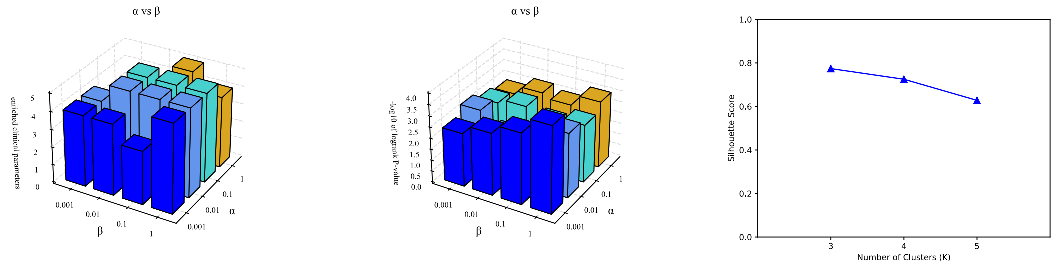

(a) (b) (c)

Figure S5. CESC-Cervical Squamous Cell Carcinoma and Endocervical Adenocarcinoma (a)Sensitivity analysis of enriched clinical parameters for M2CGCN with respect to α and β. (b)Sensitivity analysis of survival analysis for M2CGCN with respect to α and β. (c)Silhouette score (y-axis) for different number of clusters (x-axis).

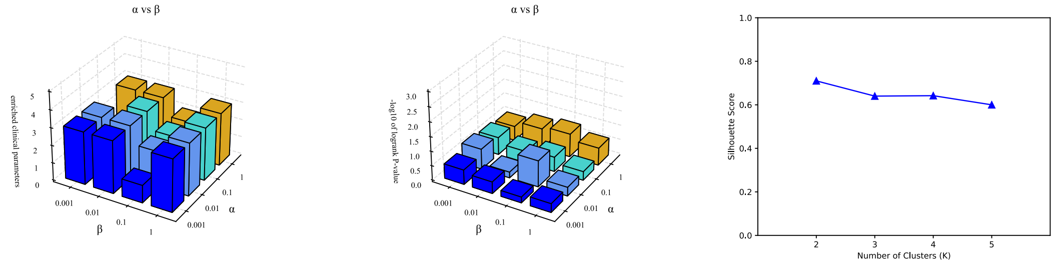

(a) (b) (c)

Figure S6. CHOL-Cholangiocarcinoma (a)Sensitivity analysis of enriched clinical parameters for M2CGCN with respect to α and β. (b)Sensitivity analysis of survival analysis for M2CGCN with respect to α and β. (c)Silhouette score (y-axis) for different number of clusters (x-axis).

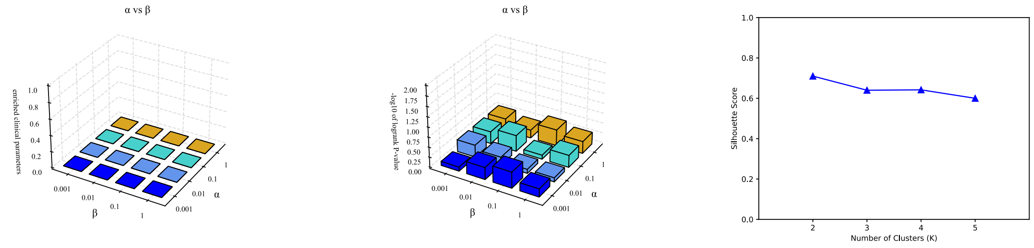

(a) (b) (c)

Figure S7. COAD-Colon Adenocarcinoma (a)Sensitivity analysis of enriched clinical parameters for M2CGCN with respect to α and β. (b)Sensitivity analysis of survival analysis for M2CGCN with respect to α and β. (c)Silhouette score (y-axis) for different number of clusters (x-axis).

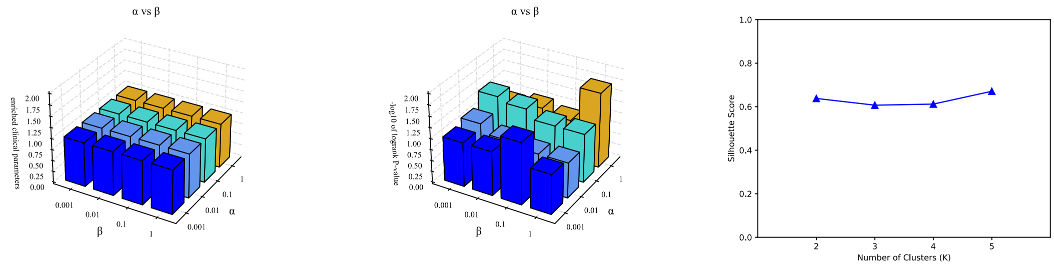

(a) (b) (c)

Figure S8. DLBC-Lymphoid Neoplasm Diffuse Large B-cell Lymphoma (a)Sensitivity analysis of enriched clinical parameters for M2CGCN with respect to α and β. (b)Sensitivity analysis of survival analysis for M2CGCN with respect to α and β. (c)Silhouette score (y-axis) for different number of clusters (x-axis).

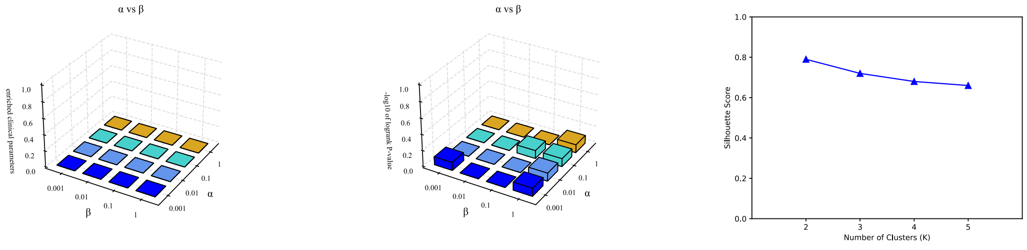

(a) (b) (c)

Figure S9. ESCA-Esophageal Carcinoma (a)Sensitivity analysis of enriched clinical parameters for M2CGCN with respect to α and β. (b)Sensitivity analysis of survival analysis for M2CGCN with respect to α and β. (c)Silhouette score (y-axis) for different number of clusters (x-axis).

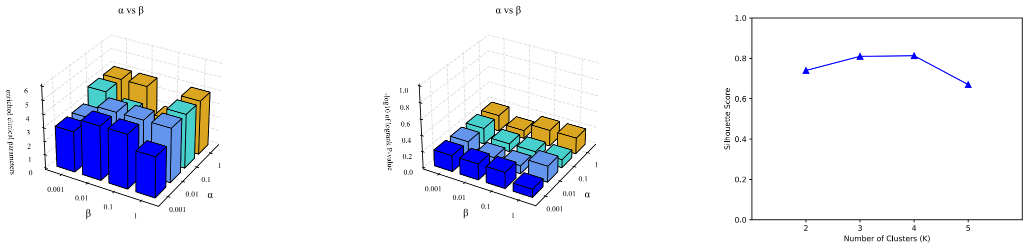

(a) (b) (c)

Figure S10. GBM-Glioblastoma Multiforme (a)Sensitivity analysis of enriched clinical parameters for M2CGCN with respect to α and β. (b)Sensitivity analysis of survival analysis for M2CGCN with respect to α and β. (c)Silhouette score (y-axis) for different number of clusters (x-axis).

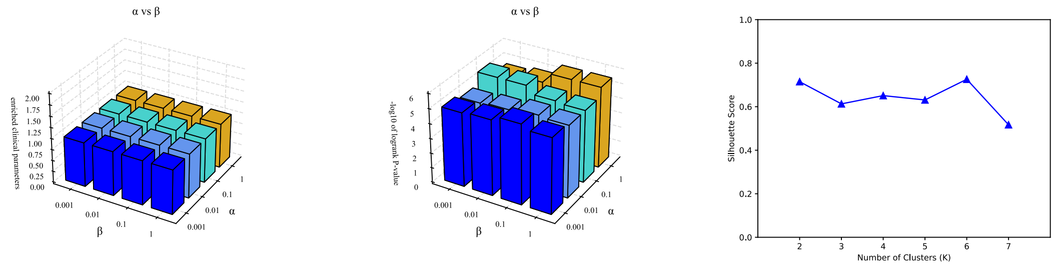

(a) (b) (c)

Figure S11. HNSC-Head and Neck Squamous Cell Carcinoma (a)Sensitivity analysis of enriched clinical parameters for M2CGCN with respect to α and β. (b)Sensitivity analysis of survival analysis for M2CGCN with respect to α and β. (c)Silhouette score (y-axis) for different number of clusters (x-axis).

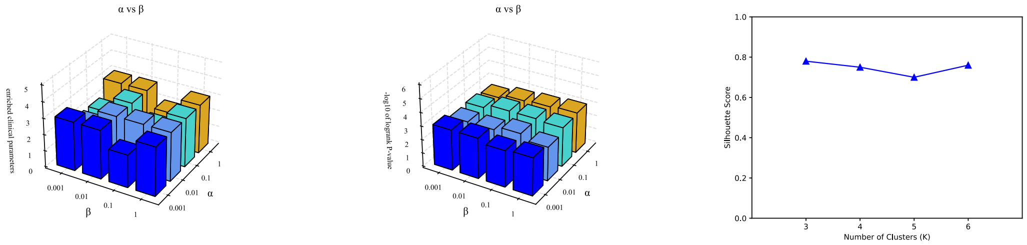

(a) (b) (c)

Figure S12. KICH-Kidney Chromophobe (a)Sensitivity analysis of enriched clinical parameters for M2CGCN with respect to α and β. (b)Sensitivity analysis of survival analysis for M2CGCN with respect to α and β. (c)Silhouette score (y-axis) for different number of clusters (x-axis).

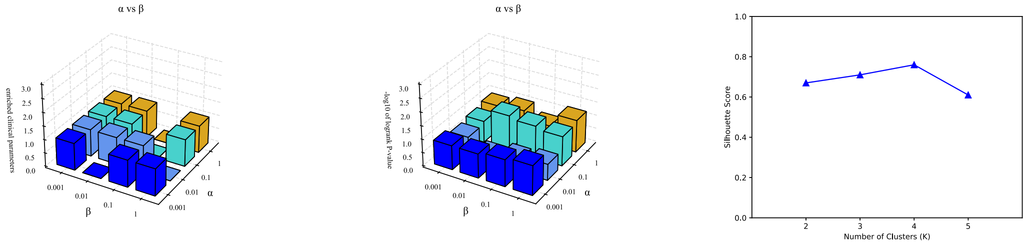

(a) (b) (c)

Figure S13. KIRC-Kidney Renal Clear Cell Carcinoma (a)Sensitivity analysis of enriched clinical parameters for M2CGCN with respect to α and β. (b)Sensitivity analysis of survival analysis for M2CGCN with respect to α and β. (c)Silhouette score (y-axis) for different number of clusters (x-axis).

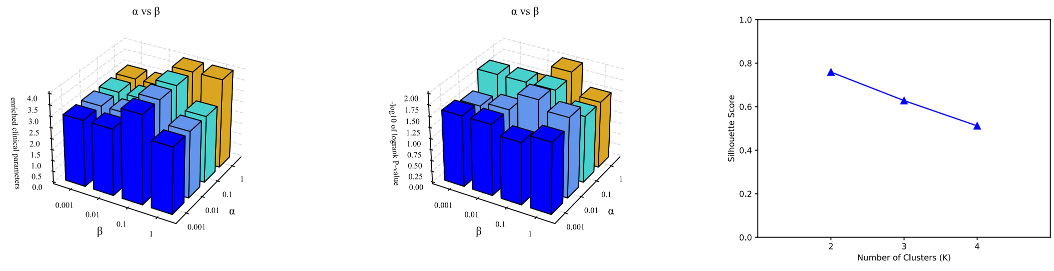

(a) (b) (c)

Figure S14. KIRP-Kidney Renal Papillary Cell Carcinoma (a)Sensitivity analysis of enriched clinical parameters for M2CGCN with respect to α and β. (b)Sensitivity analysis of survival analysis for M2CGCN with respect to α and β. (c)Silhouette score (y-axis) for different number of clusters (x-axis).

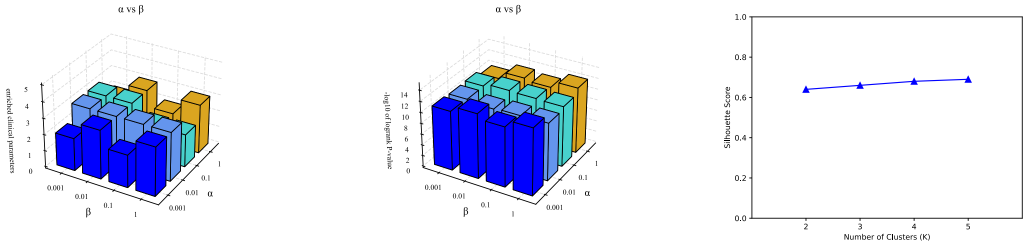

(a) (b) (c)

Figure S15. LGG-Brain Lower Grade Glioma (a)Sensitivity analysis of enriched clinical parameters for M2CGCN with respect to α and β. (b)Sensitivity analysis of survival analysis for M2CGCN with respect to α and β. (c)Silhouette score (y-axis) for different number of clusters (x-axis).

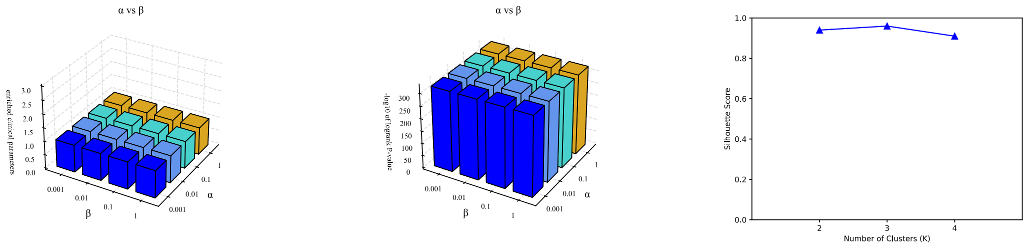

(a) (b) (c)

Figure S16. LIHC-Liver Hepatocellular Carcinoma (a)Sensitivity analysis of enriched clinical parameters for M2CGCN with respect to α and β. (b)Sensitivity analysis of survival analysis for M2CGCN with respect to α and β. (c)Silhouette score (y-axis) for different number of clusters (x-axis).

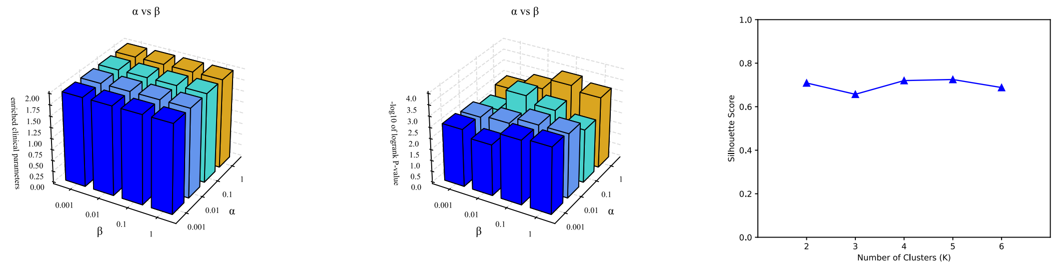

(a) (b) (c)

Figure S17. LUAD-Lung Adenocarcinoma (a)Sensitivity analysis of enriched clinical parameters for M2CGCN with respect to α and β. (b)Sensitivity analysis of survival analysis for M2CGCN with respect to α and β. (c)Silhouette score (y-axis) for different number of clusters (x-axis).

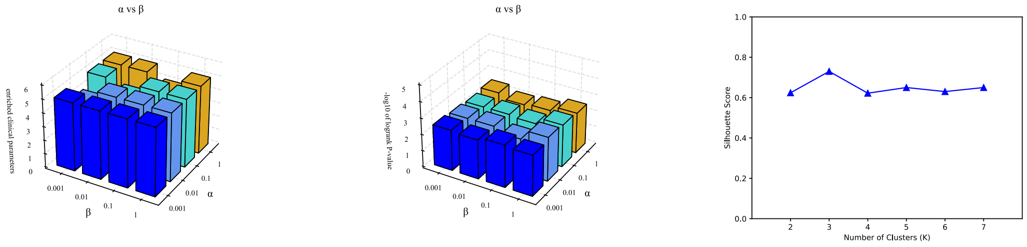

(a) (b) (c)

Figure S18. LUSC-Lung Squamous Cell Carcinoma (a)Sensitivity analysis of enriched clinical parameters for M2CGCN with respect to α and β. (b)Sensitivity analysis of survival analysis for M2CGCN with respect to α and β. (c)Silhouette score (y-axis) for different number of clusters (x-axis).

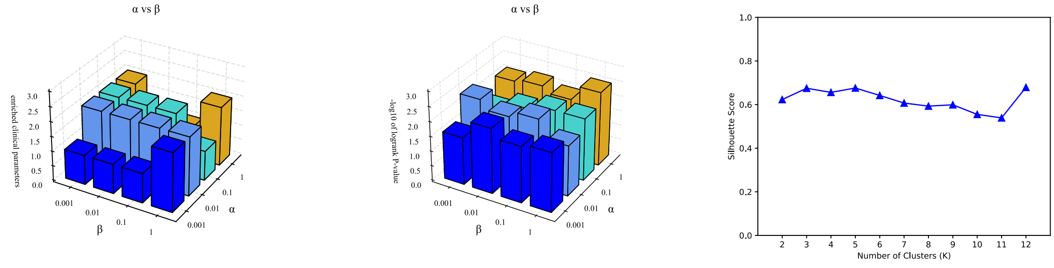

(a) (b) (c)

Figure S19. MESO-Mesothelioma (a)Sensitivity analysis of enriched clinical parameters for M2CGCN with respect to α and β. (b)Sensitivity analysis of survival analysis for M2CGCN with respect to α and β. (c)Silhouette score (y-axis) for different number of clusters (x-axis).

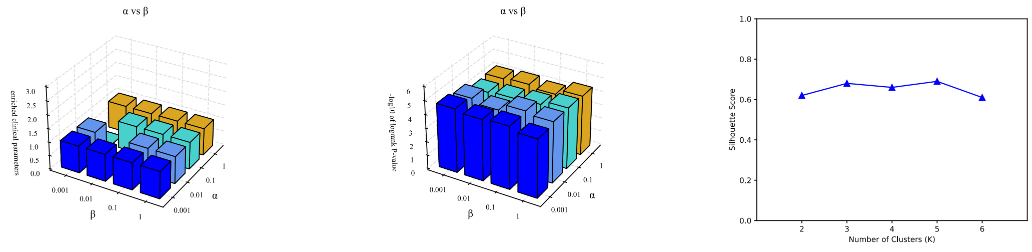

(a) (b) (c)

Figure S20. OV-Ovarian Serous Cystadenocarcinoma (a)Sensitivity analysis of enriched clinical parameters for M2CGCN with respect to α and β. (b)Sensitivity analysis of survival analysis for M2CGCN with respect to α and β. (c)Silhouette score (y-axis) for different number of clusters (x-axis).

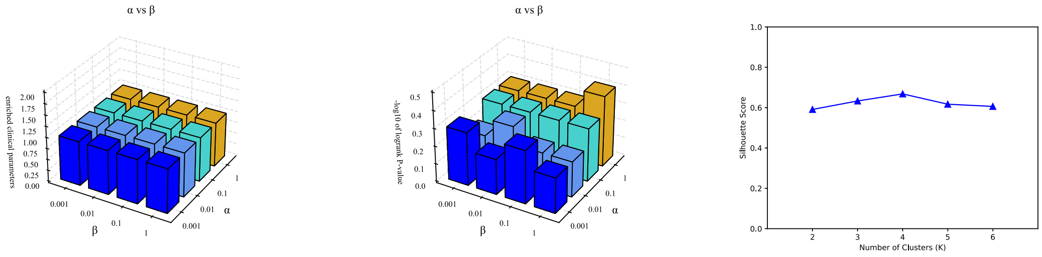

(a) (b) (c)

Figure S21. PAAD-Pancreatic Adenocarcinoma (a)Sensitivity analysis of enriched clinical parameters for M2CGCN with respect to α and β. (b)Sensitivity analysis of survival analysis for M2CGCN with respect to α and β. (c)Silhouette score (y-axis) for different number of clusters (x-axis).

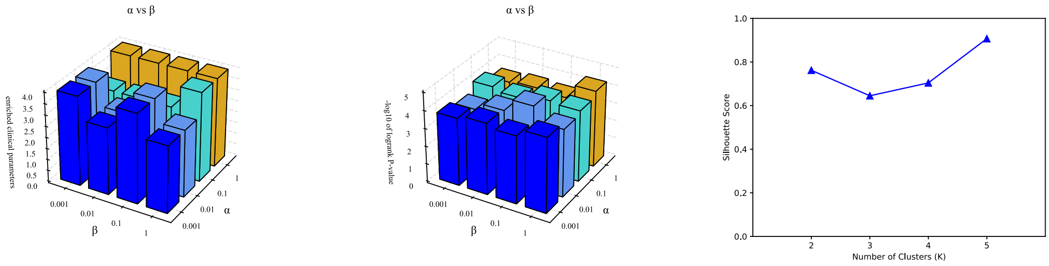

(a) (b) (c)

Figure S22. PCPG-Pheochromocytoma and Paraganglioma (a)Sensitivity analysis of enriched clinical parameters for M2CGCN with respect to α and β. (b)Sensitivity analysis of survival analysis for M2CGCN with respect to α and β. (c)Silhouette score (y-axis) for different number of clusters (x-axis).

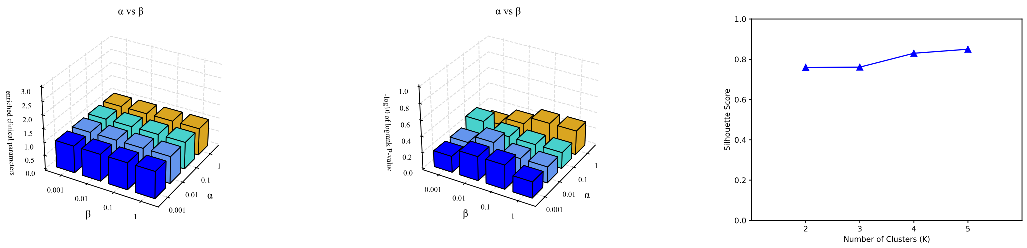

(a) (b) (c)

Figure S23. PRAD-Prostate Adenocarcinoma (a)Sensitivity analysis of enriched clinical parameters for M2CGCN with respect to α and β. (b)Sensitivity analysis of survival analysis for M2CGCN with respect to α and β. (c)Silhouette score (y-axis) for different number of clusters (x-axis).

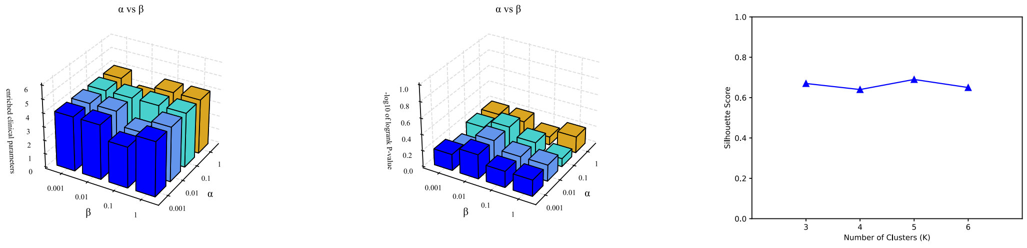

(a) (b) (c)

Figure S24. READ-Rectum Adenocarcinoma (a)Sensitivity analysis of enriched clinical parameters for M2CGCN with respect to α and β. (b)Sensitivity analysis of survival analysis for M2CGCN with respect to α and β. (c)Silhouette score (y-axis) for different number of clusters (x-axis).

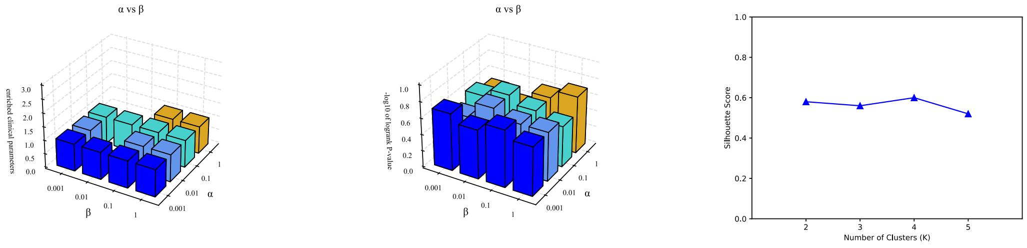

(a) (b) (c)

Figure S25. SKCM-Skin Cutaneous Melanoma (a)Sensitivity analysis of enriched clinical parameters for M2CGCN with respect to α and β. (b)Sensitivity analysis of survival analysis for M2CGCN with respect to α and β. (c)Silhouette score (y-axis) for different number of clusters (x-axis).

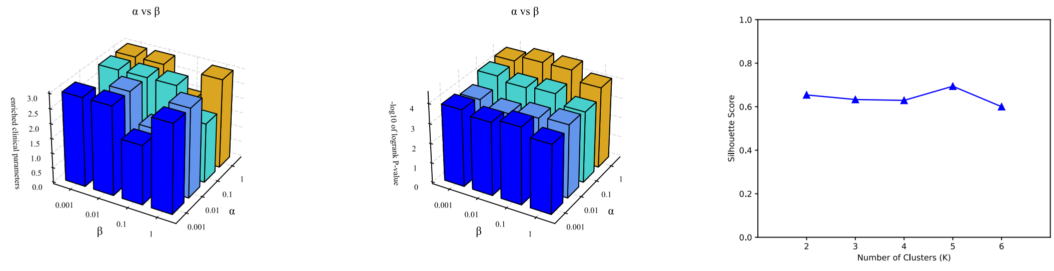

(a) (b) (c)

Figure S26. SARC-Sarcoma (a)Sensitivity analysis of enriched clinical parameters for M2CGCN with respect to α and β. (b)Sensitivity analysis of survival analysis for M2CGCN with respect to α and β. (c)Silhouette score (y-axis) for different number of clusters (x-axis).

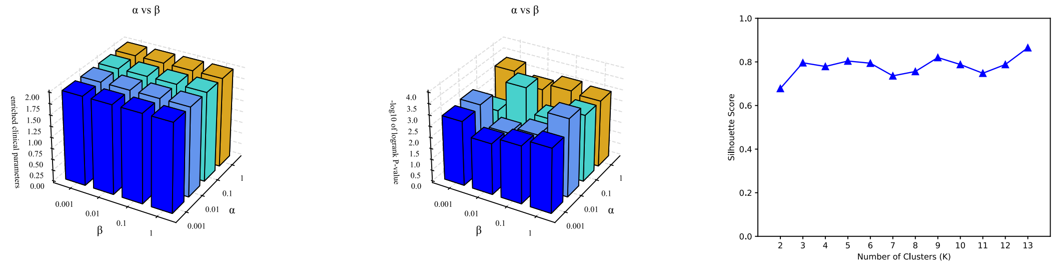

(a) (b) (c)

Figure S27. STAD-Stomach Adenocarcinoma (a)Sensitivity analysis of enriched clinical parameters for M2CGCN with respect to α and β. (b)Sensitivity analysis of survival analysis for M2CGCN with respect to α and β. (c)Silhouette score (y-axis) for different number of clusters (x-axis).

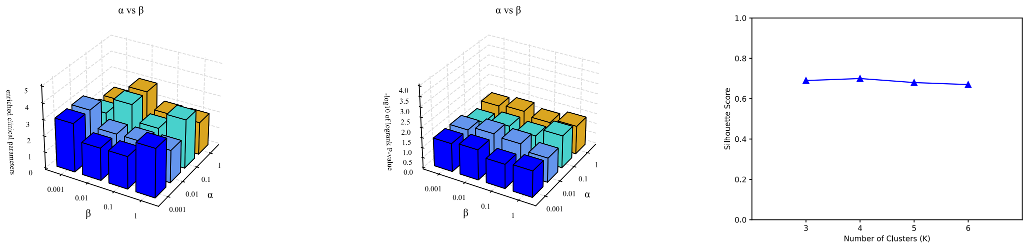

(a) (b) (c)

Figure S28. TGCT-Testicular Germ Cell Tumors (a)Sensitivity analysis of enriched clinical parameters for M2CGCN with respect to α and β. (b)Sensitivity analysis of survival analysis for M2CGCN with respect to α and β. (c)Silhouette score (y-axis) for different number of clusters (x-axis).

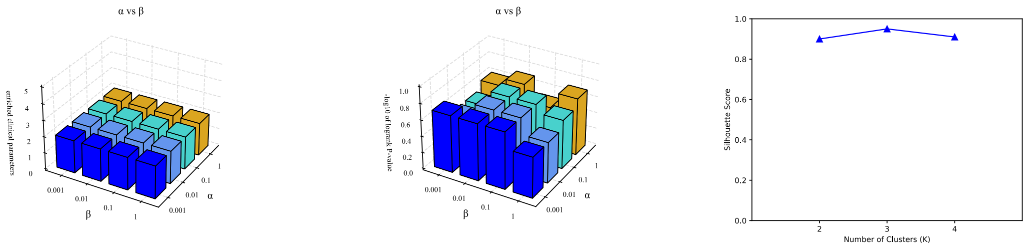

(a) (b) (c)

Figure S29. THCA-Thyroid Carcinoma (a)Sensitivity analysis of enriched clinical parameters for M2CGCN with respect to α and β. (b)Sensitivity analysis of survival analysis for M2CGCN with respect to α and β. (c)Silhouette score (y-axis) for different number of clusters (x-axis).

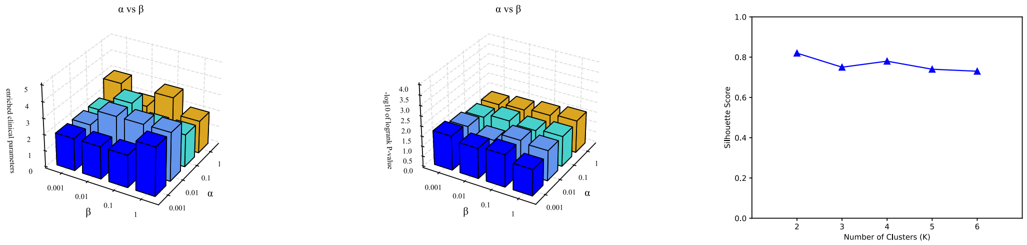

(a) (b) (c)

Figure S30. THYM-Thymoma (a)Sensitivity analysis of enriched clinical parameters for M2CGCN with respect to α and β. (b)Sensitivity analysis of survival analysis for M2CGCN with respect to α and β. (c)Silhouette score (y-axis) for different number of clusters (x-axis).

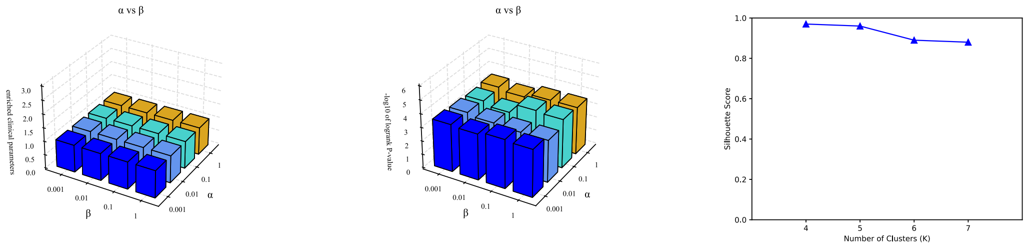

(a) (b) (c)

Figure S31. UCEC-Uterine Corpus Endometrial Carcinoma (a)Sensitivity analysis of enriched clinical parameters for M2CGCN with respect to α and β. (b)Sensitivity analysis of survival analysis for M2CGCN with respect to α and β. (c)Silhouette score (y-axis) for different number of clusters (x-axis).

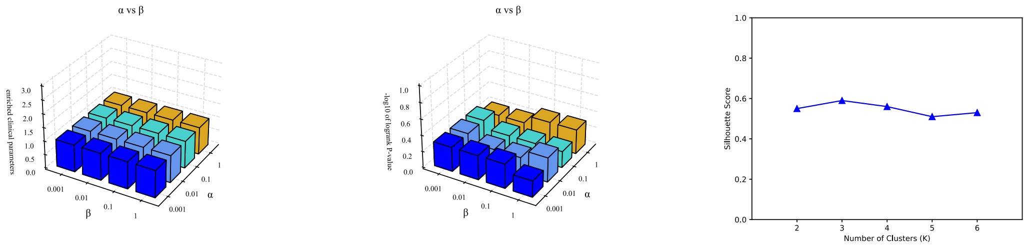

(a) (b) (c)

Figure S32. UCS-Uterine Carcinosarcoma (a)Sensitivity analysis of enriched clinical parameters for M2CGCN with respect to α and β. (b)Sensitivity analysis of survival analysis for M2CGCN with respect to α and β. (c)Silhouette score (y-axis) for different number of clusters (x-axis).

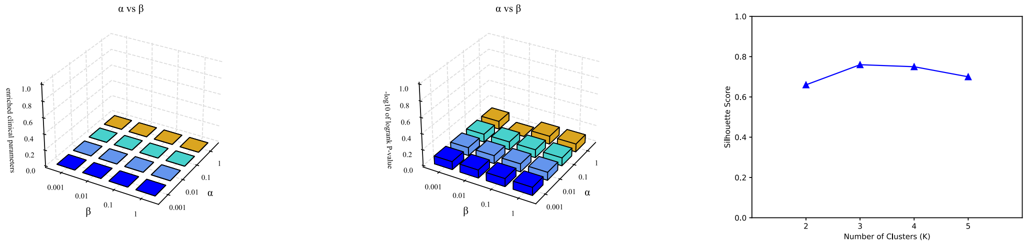

(a) (b) (c)

Figure S33. UVM-Uveal Melanoma (a)Sensitivity analysis of enriched clinical parameters for M2CGCN with respect to α and β. (b)Sensitivity analysis of survival analysis for M2CGCN with respect to α and β. (c)Silhouette score (y-axis) for different number of clusters (x-axis).

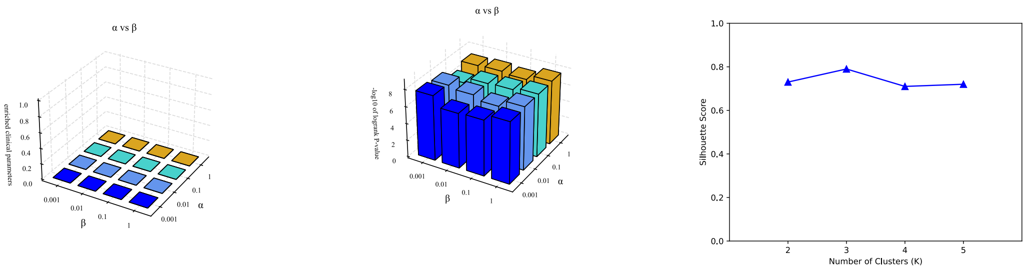

(a) (b) (c)

Figure S34. METABRIC-Molecular Taxonomy of Breast Cancer International Consortium (a)Sensitivity analysis of enriched clinical parameters for M2CGCN with respect to α and β. (b)Sensitivity analysis of survival analysis for M2CGCN with respect to α and β. (c)Silhouette score (y-axis) for different number of clusters (x-axis).

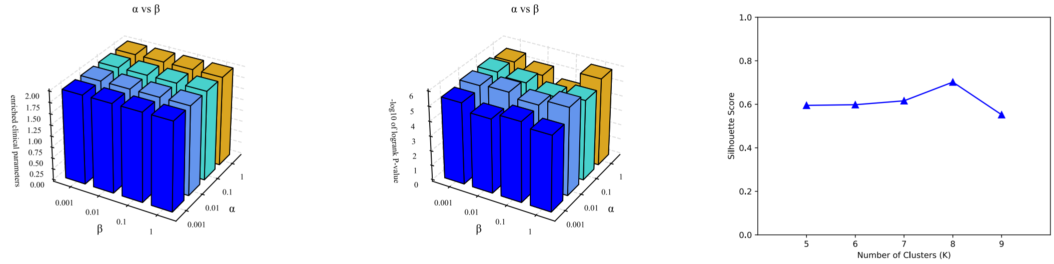

(a) (b) (c)
